# LBD-PointNet++: a point cloud segmentation network for phenotypic trait extraction of broccoli seedlings

**DOI:** 10.3389/fpls.2026.1835285

**Published:** 2026-07-31

**Authors:** Haoqi Wang, Baoqiang Yin, Siying Liu, Yuanyu Xia, Ziyi Zhang, Hongbin Wu, Xiuqing Fu

**Affiliations:** College of Engineering, Nanjing Agricultural University, Nanjing, China

**Keywords:** 3D point cloud, broccoli seedlings, large kernel attention, LBD-PointNet++, phenotyping, salt stress, semantic segmentation

## Abstract

Broccoli is a globally significant vegetable, but climate change and soil salinization increasingly threaten its productivity. Precise seedling phenotyping is essential for selecting salt-tolerant germplasm, yet traditional manual methods are labor-intensive and error-prone. This study develops LBD-PointNet++, an optimized 3D point cloud semantic segmentation model for automated phenotypic parameter extraction of broccoli seedlings at the germination and early developmental phase under salt stress. High-fidelity 3D point clouds were reconstructed from a precision three-view imaging system using Structure from Motion (SfM) algorithms. LBD-PointNet++ introduces three core optimizations: (1) a Large Kernel Attention (LKA) mechanism using 3D sparse decomposition to capture long-range global dependencies; (2) a Dual Uncertainty and Shape-Adaptive Sampling (DUSAS) mechanism to preserve high-frequency features of fragile stems and margins; and (3) a joint Boundary-Aware Nested Contrastive and Adaptive Varifocal Joint Loss (BNCV-Loss) to effectively isolate overlapping leaves. Experimental results demonstrate superior performance, achieving an overall mean Intersection over Union (mIoU) of 88.07% across all three categories (Leaf, Stem, and Pot) and a Mean F1-score of 93.48%. Compared to state-of-the-art Transformer architectures like PTv3, LBD-PointNet++ achieves higher accuracy with less than 6% of the parameter volume and over twofold faster inference speed. Furthermore, dynamic monitoring across NaCl gradients (0–250 mmol/L) revealed a potential non-linear threshold effect, identifying 100 mmol/L as a preliminary phenotypic threshold under these conditions. Beyond this threshold, growth inhibition intensified rapidly; At 250 mmol/L, plant height decreased by 54.43% and the 3D entity volume shrank to approximately one-fifth of the control group. In summary, LBD-PointNet++ provides a high-efficiency solution for phenotypic identification and digital breeding of salt-tolerant Brassicaceae crops.

## 1 Introduction

Plant phenotyping encompasses a suite of quantifiable characteristics—including morphology, structure, physiological traits, and growth status—arising from the dynamic interplay between genotypes and environmental factors. Accurate and high-throughput phenotypic analysis serves as the core pillar for deciphering crop stress response mechanisms and facilitating genetic improvement. Driven by the rapid integration of artificial intelligence and sensor technologies, the non-destructive, multi-dimensional, and automated acquisition of phenotypes has become a key focus area in smart agriculture research (Song et al., 2021). Phenotypic assessment of seedlings is particularly vital for breeding and seed development; in practical breeding, leveraging growth traits during the seedling phase for early screening allows for the preemptive identification of elite individuals, thereby substantially bolstering overall breeding efficiency. As a fundamental parameter for quantifying plant growth and development, rapid and precise estimation methods for plant height provide critical support for enhancing the accuracy and efficiency of crop yield forecasting (Quan et al., 2024). Plant volume constitutes a significant visual metric of growth status and biomass accumulation, offering a comprehensive reflection of growth vigor and spatial expansion capacity (Xiao et al., 2020). Leaf area expansion dictates the light interception capacity of crops and is frequently utilized as a surrogate indicator for plant growth in high-throughput phenotyping systems (Weraduwage et al., 2015).

Broccoli (Brassica oleracea L. var. italica) is a vital cruciferous vegetable crop that possesses significant nutritional and economic value; it is rich in bioactive substances like glucosinolates and phenolic compounds (Shinali et al., 2024) and contains potential anticancer agents such as indole-3-carbinol and 3,3’-diindolylmethane (Shinali et al., 2024). However, the continuous impact of climate change and deteriorating environmental conditions has led to abiotic stresses that increasingly limit plant development and degrade productivity (Sánchez-Bermúdez et al., 2022). As a primary abiotic stressor in global agriculture, salt stress significantly affects crop physiological metabolism and yield by disrupting osmotic balance and inducing oxidative stress, leading to phenotypic abnormalities such as stunted height, diminished stem diameters, and inhibited biomass accumulation (Wang et al., 2022). Recent studies indicate that morphological variations in broccoli seedlings are closely linked to the activation of internal osmoregulation and antioxidant systems (Wang et al., 2022). Given that the sensitivity of seedlings to salinity directly dictates subsequent growth trajectories and yield potential, elucidating the phenotypic response patterns of broccoli seedlings under salt stress is of theoretical and practical importance.

Traditional crop phenotypic assessment relies on manual measurements, which are labor-intensive, time-consuming, and prone to subjective errors; these methods struggle to achieve precise, multi-indicator dynamic monitoring across the temporal scales required for salt stress gradient experiments. With advancements in sensing technology and artificial intelligence, 2D phenotypic analysis utilizing Convolutional Neural Networks (CNNs) (Jiang and Li, 2020) has been widely adopted. For instance, a lightweight deep learning object detection model based on YOLOv7 was developed to accurately assess the planting quality of broccoli seedlings in the field (Zhang et al., 2024). However, when confronted with complex plant architectures or the need for simultaneous measurement of multiple phenotypic traits, 3D reconstruction offers superior utility. Due to its non-destructive nature and high resolution, 3D reconstruction has become a pivotal means of overcoming the bottlenecks of traditional phenotyping. As the core data format for 3D analysis, point clouds preserve comprehensive spatial structural information. Multi-view reconstruction techniques provide richer topological semantics than traditional 2D images when handling seedlings with complex self-occlusion features (Song et al., 2021). This allows for the authentic reflection of 3D morphological characteristics, providing a foundation for the precise extraction of parameters like plant height, stem diameter, and leaf area. Recent progress includes an automated pipeline based on multi-view stereo vision and deep learning point cloud segmentation for extracting pod and stem phenotypes in mature soybeans (Cui et al., 2025).

The rise of deep learning has provided powerful tools for efficient point cloud analysis. PointNet, the first end-to-end network to process point cloud data directly, bypassed the traditional reliance on data regularization by aggregating global features through symmetric functions, effectively enabling classification and segmentation (Qi et al., 2017a). This laid the groundwork for organ-level plant phenotyping. Derived models, such as PointNet++ (Qi et al., 2017b), have further enhanced local feature extraction by capturing spatial information across various scales through hierarchical learning frameworks. These models have shown excellent performance in the semantic and instance segmentation of plant organs like stems and leaves. For example, a method combining UAV-based 3D point clouds with an improved PointNet++ model was proposed for tobacco plant segmentation and automated phenotypic extraction (Yao et al., 2025). Similarly, the CAVF-PointNet++ model achieved a total accuracy of 96.93% and a mean Intersection over Union (mIoU) of 82.56% in sesame organ segmentation—outperforming the baseline PointNet++ by 1.72% and 3.64%, respectively—offering a robust reference for 3D phenotyping in various crops (Wei et al., 2025). Furthermore, other studies have explored integrating clustering algorithms to enhance local feature representation in PointNet-based networks, demonstrating its effectiveness in improving segmentation performance (Eisl and Halperin, 2025).

Currently, while PointNet++ based research has progressed in crops like maize, tomato, and wheat, point cloud-based phenotypic analysis for Brassicaceae seedlings under abiotic stress (particularly salt stress) remains scarce. There is a notable lack of systematic exploration using PointNet++ to quantify the impact of salinity on key phenotypic traits such as plant height, projected leaf area, and 3D volume. The complex organ structure of broccoli seedlings requires high-precision modeling to capture subtle morphological changes under stress. PointNet++ is uniquely suited to handle irregular point clouds while preserving spatial context, making it possible to capture the dynamic shifts in broccoli seedling phenotypes. Furthermore, as a functional vegetable rich in bioactive components (Zhang et al., 2023), enhancing broccoli’s salt tolerance is critical for expanding cultivation areas and stabilizing yields. High-throughput phenotyping via point cloud models can provide a fast, objective basis for the selection of salt-tolerant varieties.

This study proposes an improved semantic segmentation model, LBD-PointNet++, designed for the rapid and precise segmentation of broccoli seedling leaves and stems in salt-stressed environments. The key phenotypic parameters extracted—including plant height, projected leaf area, and 3D volume—provide core data support for assessing growth status across salinity gradients during this narrow early growth window. Our primary contributions are as follows:

1. Dataset Construction: High-resolution raw images were captured using a precision three-view imaging system, and 60 sets of high-fidelity broccoli seedling point clouds were reconstructed using SfM algorithms. Following anatomical priors, the data were categorized into “Leaf,” “Stem,” and “Pot” classes, with labels cross-validated by experts to ensure precision.
2. LBD-PointNet++ Model: An optimized segmentation framework was developed. It integrates a Large Kernel Attention mechanism via 3D sparse decomposition to capture broad global context and complex plant topologies. It introduces a Structured Sampling Mechanism driven by dual uncertainty to preserve high-frequency features of thin stems and distorted edges. Finally, a Joint Loss Function creates a sharp feature polarization effect to separate overlapping leaves and address the challenges of minor-sample categories and long-tail data distributions.
3. Automated Parameter Extraction: We provide a set of mathematical models based on geometric topology that automatically fit the cultivation substrate plane and utilize DBSCAN clustering and convex hull theory to precisely calculate plant height, projected leaf area, and 3D volume.
4. Growth Experiments under Salt Stress: Dynamic monitoring was conducted across a NaCl gradient (0 to 250 mmol/L). We identified a distinct non-linear response: a “low-concentration stimulation effect” at 100 mmol/L, followed by a rapid intensification of growth inhibition beyond this threshold. Under high salinity (250 mmol/L), plant height decreased by 54.43% and volume shrank to approximately one-fifth compared to the control group.

## 2 Materials and methods

### 2.1 Experimental equipment and experimental design

**Figure 1:**
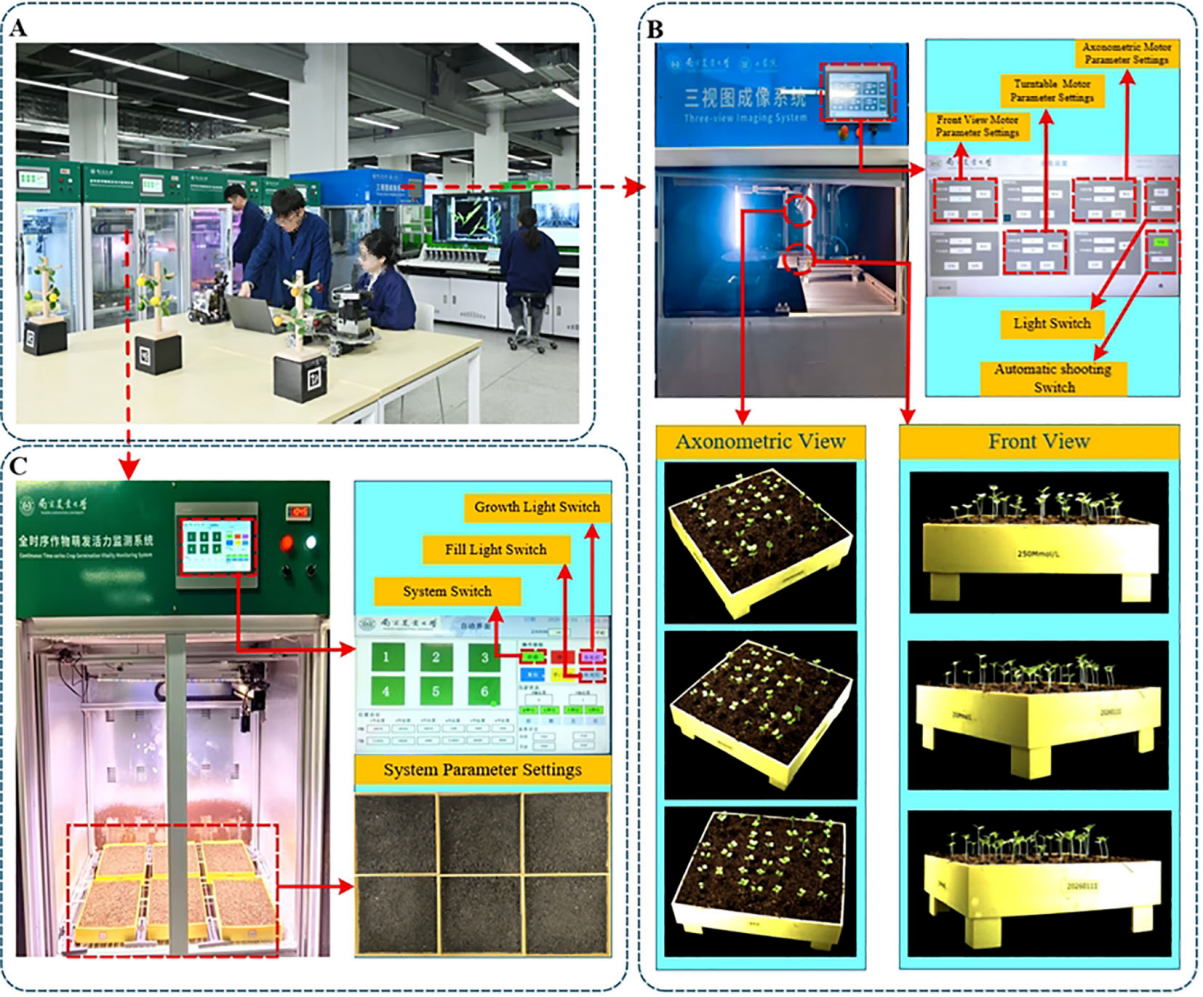
**(A)** Experimental scenario; **(B)** three-view imaging system; **(C)** cultivation of broccoli seedlings.

In this study, we utilized an integrated crop growth and phenotypic monitoring system, as illustrated in **Figure 1**, which consists of a deeply integrated environmental control cultivation module and a precision orbital image acquisition module. The core of the cultivation module is a fully enclosed, controlled-environment chamber designed to provide a highly stable physical space and microclimate for crop development. The interior of the cultivation box employs perforated partitions for hierarchical spatial utilization; the upper layer houses six sets of specialized acrylic culture trays to support high-density broccoli seedling cultivation, while the lower layer is designed as a deionized water storage area to ensure the purity of the critical water supply throughout the experiment. To achieve precise regulation of the growth environment, the system integrates an embedded PTC (Positive Temperature Coefficient) hot air circulation control scheme. This setup utilizes a TP-100 thermocouple sensor mounted on the chamber’s side wall to monitor temperature field variations in real-time. When the monitored temperature falls below a pre-established threshold, the control logic automatically triggers the hot air circulation to compensate for thermal loss until the steady-state upper limit is reached, thereby maintaining high thermal stability across fluctuating experimental cycles. User interaction and parameter configuration are facilitated via an industrial-grade touch screen installed at the top of the box, complemented by a power control matrix integrated on the upper right side, allowing researchers to intervene in real-time regarding system power, LED supplemental lighting arrays, and temperature regulation logic.

At the phenotypic perception level, a precision three-view imaging subsystem was employed. The mechanical framework of this system is constructed from high-strength industrial aluminum profiles, providing a high-rigidity mounting foundation for the complex optical precision components. The heart of the imaging unit is a high-resolution HIKVISION RGB industrial camera (model MV-CS200-10GC), capable of capturing ultra-clear raw images with a resolution of 5472×3648 pixels, thereby providing a high-quality data source for the subsequent generation of high-precision 3D point clouds. To optimize image quality and eliminate perspective distortion, the camera is equipped with a specially customized telecentric lens. Additionally, a multi-angle LED cold light source array is integrated within the system to provide uniform illumination compensation for seedlings at various growth stages. Flexibility and multi-dimensionality in image acquisition are guaranteed by high-precision motion-drive components. The system utilizes a ball screw module to perform micron-level stepping adjustments of the camera’s spatial position, meeting the imaging requirements of different observation heights. The acquisition platform is further equipped with an electric laser rotary table at its base, which carries the culture trays for fixed-angle rotation. In synchronization with the industrial camera, this setup enables the automated non-destructive acquisition of multi-view images, including front, side, top, and axonometric views. The entire data collection process is centrally orchestrated by an integrated customized control panel, ensuring a high degree of temporal and spatial consistency between camera exposure and rotary table displacement. This rigorous synchronization establishes a robust hardware foundation for sub-millimeter precision 3D point cloud reconstruction using algorithms such as Colmap.

### 2.2 Data collection and preprocessing

**Figure 2:**
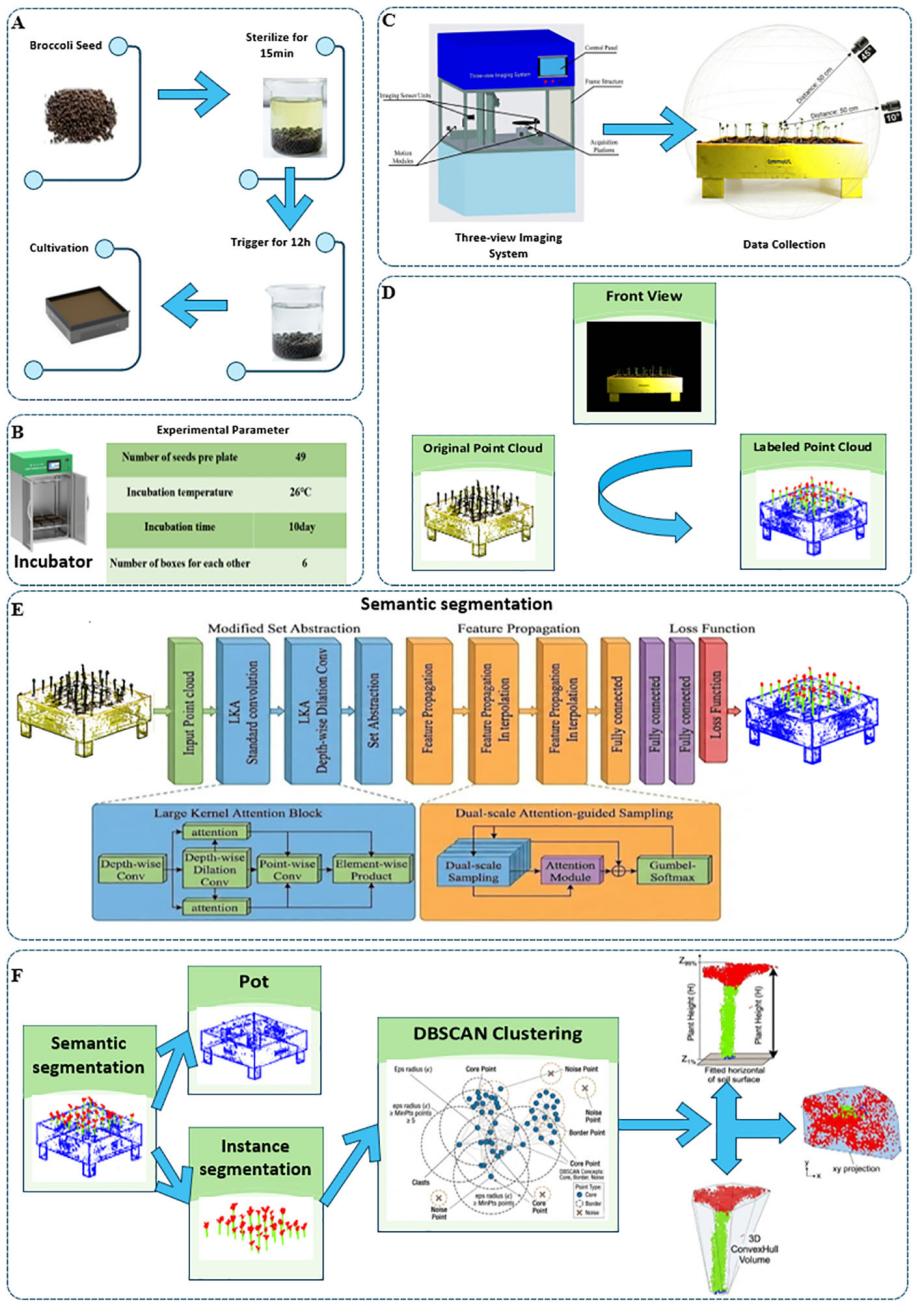
**(A)** Broccoli seed pretreatment and sowing experimental workflow; **(B)** controlled-environment cultivation chamber and its core operational parameters; **(C)** three-view imaging system and automated multi-perspective data acquisition protocol; **(D)** 3D reconstruction of raw point clouds and semantic labeling process; **(E)** global architecture of the LBD-PointNet++ point cloud semantic segmentation network; **(F)** individual plant clustering and key phenotypic parameter extraction workflow.

To accurately characterize the phenotypic traits of broccoli during the seedling stage following the comprehensive workflow shown in **Figure 2**, 588 uniform and morphologically intact seeds were carefully selected. The seeds were initially sterilized in a sodium hypochlorite solution for 15 minutes to effectively eliminate surface-borne pathogens, thereby mitigating the risk of seedling diseases and enhancing germination uniformity. This was followed by 2–3 washes with deionized water to remove residual disinfectant and prevent chemical damage to the seed embryo. Subsequently, the seeds were soaked in deionized water at 30°C for 12 hours to soften the seed coat, facilitate hydration, and initiate pre-germination metabolic activities, thus improving the germination rate. Finally, the broccoli seeds were precision-sown in acrylic culture trays with a side length of 25 cm for growth and cultivation. To simulate the population competition and occlusion effects characteristic of high-density planting environments, each tray followed a strict 7×7 matrix arrangement (totaling 49 seeds per tray). To capture critical geometric features of plant development, image acquisition was concentrated during the primary seedling stage, utilizing a three-view imaging system for 360° automated data capture. During the 3D spatial modeling phase, a multi-view Structure from Motion (SfM) algorithm was employed within the open-source Colmap framework to perform spatial alignment and feature matching on the captured array video streams. To establish a standardized metric reference, we utilized the known physical dimension of the acrylic culture tray (exactly 25.0 cm) as a ground-truth scale anchor. A rigid 3D Euclidean transformation was applied to calibrate the dimensionless relative coordinates generated by Colmap into true-to-scale metric units (centimeters). By integrating multi-dimensional visual information from orthogonal and axonometric perspectives, the system successfully reconstructed high-fidelity raw point cloud models. Following quality assessment and redundancy elimination, 60 representative high-quality 3D datasets were ultimately selected and stored in.ply format.

To provide the model with semantic information, CloudCompare software was used to perform refined manual annotation of the point clouds. The annotation task strictly followed anatomical prior knowledge, classifying point cloud units into three semantic categories: “Leaf,” “Stem,” and “Pot”. For high-occlusion regions and complex stem-leaf junctions, a consistency determination strategy based on local geometric neighborhoods was implemented; specifically, points were classified according to the dominant local anatomical structure rather than isolated coordinates. Statistical analysis of the annotated dataset revealed a severe geometric class imbalance. Across both the training and test sets, the average point distribution is highly skewed: the cultivation container (‘Pot’) accounts for the vast majority at approximately 58.2%, followed by ‘Leaf’ at 38.5%, while the structurally critical ‘Stem’ constitutes a mere 3.3% of the total point cloud. Furthermore, the dataset underwent cross-validation by two professionals, with a conservative labeling approach applied to classification ambiguities to minimize the risk of noise propagation during the model training phase. Considering the computational resource limitations of high-throughput phenotyping platforms, selecting an appropriate downsampling scale is critical. Crucially, *N* = 4,096 does not represent the input point count of the entire 49-plant culture tray but rather denotes the standardized input size for an individual cropped plant patch during the organ-level semantic segmentation stage. The original raw point cloud of an entire culture tray reconstructed via SfM contains over 150,000 points. The processing workflow operates hierarchically: First, the high-density tray-level point cloud is partitioned into 49 independent physical units (individual plants) using automated substrate plane fitting and DBSCAN clustering. Second, each isolated single-plant point cloud is downsampled and normalized to *N* = 4,096 points. Consequently, each individual plant stem contains approximately 300 to 400 points, preserving high structural resolution for fragile stems. This higher relative proportion (7.3%–9.8% within the 4,096 patch) compared to the global dataset distribution (3.3%) stems from the hierarchical preprocessing workflow, which isolates single-plant regions and discards the vast, uninformative flat surfaces of the surrounding cultivation container (“Pot”).

To rigorously determine the optimal configuration, empirical evaluation was conducted by systematically evaluating input point counts across different scales (N = 4,096, 8,192, and 16,384). Experimental results demonstrated that elevating *N* to 8,192 and 16,384 yielded only marginal foreground mIoU gains of +0.68% and +0.95%, respectively. Conversely, the model’s inference latency escalated disproportionately from 610.12 ms (at *N* = 4,096) to 1150.4 ms and 2435.8 ms, accompanied by critical GPU memory overhead. Supported by the proposed DUSAS mechanism—which dynamically prioritizes structurally critical boundary points over homogeneous leaf interiors during downsampling—the LBD-PointNet++ robustly extracts high-fidelity topological skeletons even in highly sparse regimes. Consequently, these systematic control experiments definitively prove that *N* = 4,096 represents the optimal operational parameter, securing an favorable balance between sub-millimeter organ precision and computational runtime efficiency. Regarding the dataset composition and partitioning logic, the experiment utilized exactly 12 independent physical trays (6 NaCl concentrations × 2 independent biological replicates). Over the growth period, non-destructive 3D imaging was performed at 5 developmental time points (36h, 60h, 84h, 108h, and 132h) for these 12 physical trays, yielding a total of 60 distinct time-series point cloud datasets (scans). Each individual culture tray was treated as a physically independent observation unit to avoid “data leakage” issues caused by the temporal correlation of the same plant at different growth nodes. Ultimately, the dataset partitioning was re-implemented using a strict tray-disjoint split mechanism at the physical tray level to eliminate any potential temporal correlation leakage. Specifically, the 12 independent physical trays were partitioned using a stratified approach based on salt concentration: 10 physical trays (and all their corresponding 50 temporal scans) were assigned to the training set, while the remaining 2 independent physical trays (and all their corresponding 10 temporal scans) were reserved exclusively for the test set. This guarantees that no physical tray contributes scans to both sets.

### 2.3 LBD-PointNet++

**Figure 3:**
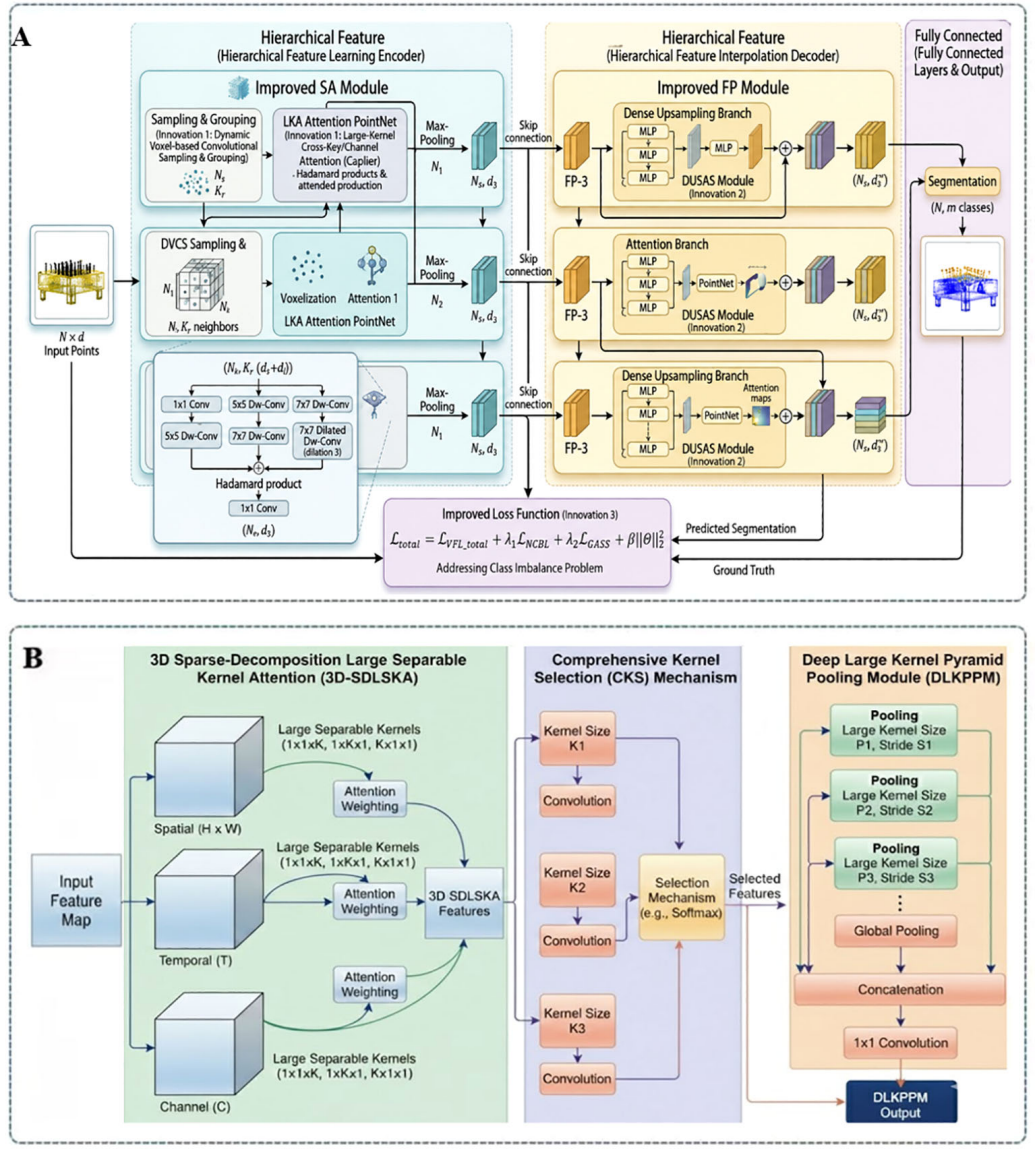
**(A)** Global architecture of LBD-PointNet++; **(B)** the LKA architecture;.

We propose a point cloud semantic segmentation network based on a hierarchical feature learning architecture, designed to achieve high-precision segmentation in complex scenarios by enhancing local geometric perception and multi-scale feature fusion. The global architecture of this model is illustrated in **Figure 3A**.

The whole framework is composed of three organically coupled parts spanning feature encoding, decoding and model optimization: the feature extraction branch adopts improved SA embedded with DVCS and LKA; the decoding branch is built upon DUSAS-optimized FP module; a compound multi-term loss is introduced in training for mitigating class imbalance and boundary ambiguity.

In the feature extraction stage, the model utilizes a modified Set Abstraction (SA) module, which integrates Dual-Vector Coordinate Selection (DVCS) sampling and grouping strategies alongside a Large Kernel Attention (LKA)-enhanced PointNet++ framework. As shown in **Figure 3B**, the core of this stage involves the introduction of the LKA system. Through the 3D Sparsely Decomposed Large Separable Kernel Attention (3D-SDLSKA) mechanism, paired with a Comprehensive Kernel Selection (CKS) mechanism, the model can adaptively capture spatial and channel features across various scales. Subsequently, a Deep Large-Kernel Pyramid Pooling Module (DLKPPM) is employed to further strengthen the extraction of global contextual information, significantly improving the model’s ability to recognize elongated or irregular structures.

**Figure 4:**
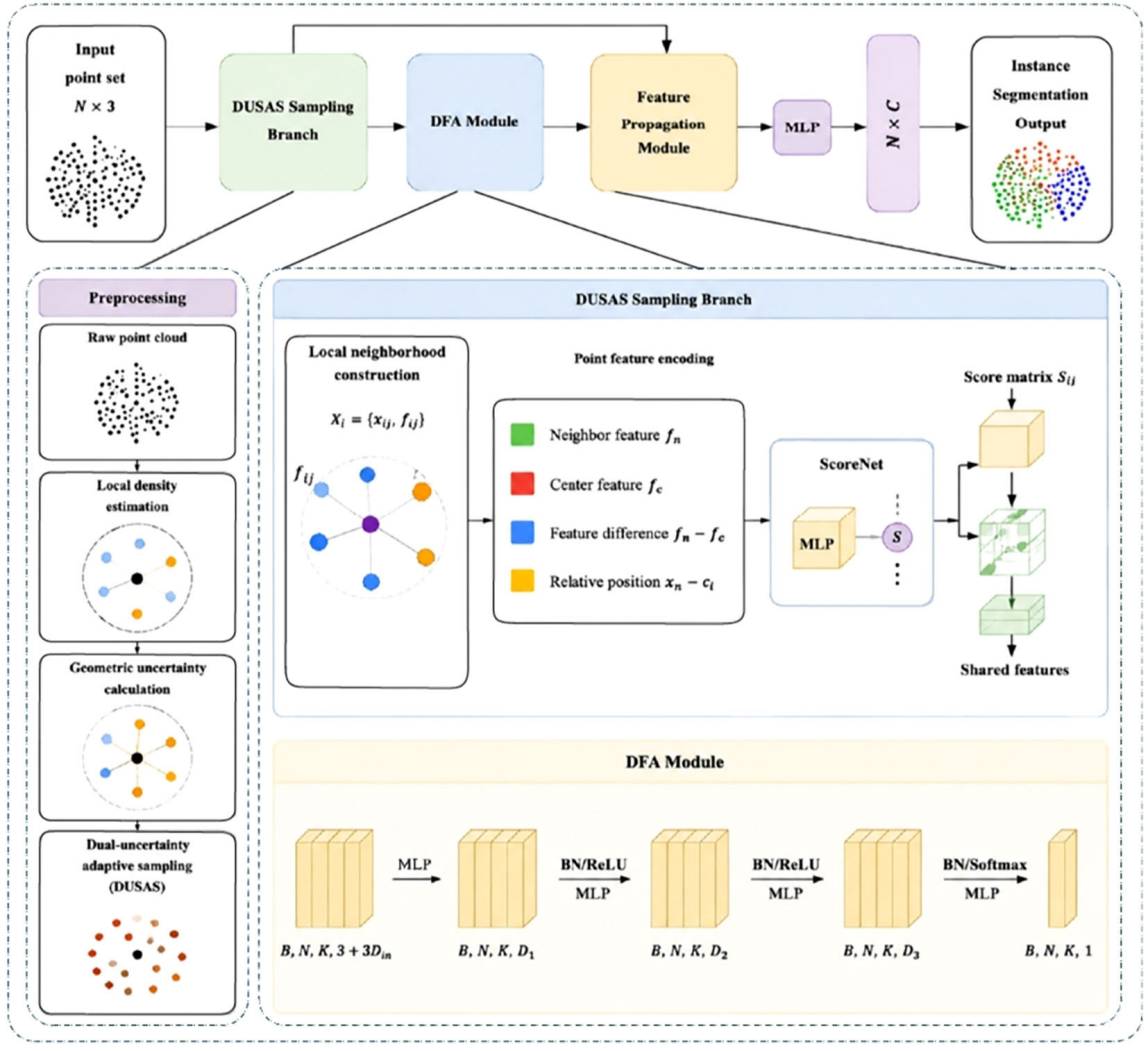
The DUSAS architecture.

In the decoding and output stage, the model incorporates a modified Feature Propagation (FP) module, centered around the DUSAS architecture. As depicted in **Figure 4**, this architecture achieves a deep fusion of dense upsampling branches and attention branches through DU-FPS sampling and a ScoreNet-based Decoupled Feature Aggregation (DFA) mechanism. This design effectively resolves feature sparsity issues during the upsampling process and enhances the representational precision of point cloud boundaries. To further improve the model’s robustness, a modified loss function is utilized during training. By combining 3D Adaptive Varifocal Loss, Nested Contrastive Boundary Loss, and Geometric Structural Similarity Loss (as shown at the bottom of **Figure 3A**), the model optimizes specifically for class imbalance, thereby significantly increasing segmentation accuracy for complex edges and minor-sample categories.

#### 2.3.1 LKA

In complex 3D unstructured point cloud environments—such as broccoli seedlings undergoing geometric distortions due to interference from varying concentrations of salt stress—the standard PointNet++ architecture faces a topological contradiction between local microscopic features and global contextual dependencies due to its use of fixed and isotropic ball queries. If massive large-kernel feature aggregation is directly introduced in 3D space to capture long-range dependencies, the number of neighboring points will undergo a rapid growth proportional to the cubic volume, scaled as *O*(*K*^3^), as the receptive field boundaries expand, leading to severe computational bottlenecks and memory overflows. To overcome this theoretical limitation, we develop a Large Kernel Attention (LKA) system tailored for 3D unordered point clouds, drawing on the mathematical underpinnings of three-dimensional sparse large-kernel architectures used in spatial feature mapping (Li et al., 2024). By deeply restructuring the feature extraction backbone, this system mathematically decouples spatial complexity. It is primarily composed of three core components: 3D Sparsely Decomposed Large Separable Kernel Attention (3D-SDLSKA), a Comprehensive Kernel Selection (CKS) mechanism, and a Deep Large-Kernel Pyramid Pooling Module (DLKPPM). Compared to traditional 3×3 convolutions, large-kernel convolutions possess a stronger global inductive bias for capturing the overall structural architecture of plants (Li et al., 2024).

First, addressing the high computational complexity inherent in 3D large-kernel computation, we derived the 3D-SDLSKA. Utilizing high-dimensional tensor decomposition principles, this mechanism strictly decouples a massive 3D receptive field into a minute, fine-grained local core neighborhood and three extremely thin, discrete, strip-like dilated neighborhoods extending orthogonally along the Cartesian axes (X, Y, and Z). Given the local features of the input point cloud *F_in_*, the network first extracts microscopic structural features *F_local_*, which preserve high-frequency boundary details, through dense local core aggregation. Subsequently, along the three independent axial directions of X, Y, and Z, a dilation stride factor *d* is introduced to facilitate sparse-leap long-range graph information transmission, as formulated in Equation 1:

(1) $$F_{dir}^{\left(i\right)}=\underset{pk\in N_{dir-strip}\left(pi\right)}{\sum }\alpha_{i,k}^{dir}\cdot V_{dir}\left(F_{local}^{\left(k\right)}\right),\;dir\in \left\{X,Y,Z\right\}$$

Where ${\text{α}}_{i,k}^{dir}$ represents the dynamic semantic correlation weights computed under the multi-head attention mechanism. By executing an element-wise summation and fusion of the macro-sparse features across three dimensions, the global structural features $F_{global}^{\left(i\right)}=F_{X-dir}^{\left(i\right)}+F_{Y-dir}^{\left(i\right)}+F_{Z-dir}^{\left(i\right)}$, which possess an expanded anisotropic 3D receptive field—are reconstructed. This topological decoupling procedure effectively reduces the computational complexity of feature aggregation with respect to the ultra-large kernel extent (*K_l_*), transitioning from the prohibitive cubic computational complexity growth *O*(*K*^3^) inherent to standard dense 3D convolutions to an asymptotically linear form $O\left(K_{s}^{3}+3K_{l}+1\right)$. Since the local core neighborhood size *K_s_* is a fixed, minor constant, the cubic term $K_{s}^{3}$ remains static and does not scale with the expansion of the receptive field, enabling a lossless, linear-scaling extension of the receptive field breadth with respect to *K_l_*.

Secondly, given that various plant organs exhibit significant morphological disparities and intra-class scale variances under different salt stress gradients, we integrated a Comprehensive Kernel Selection (CKS) mechanism to perform adaptive, dynamic gated fusion of multi-scale features. The CKS module conducts joint spatial and channel-wise statistical analysis on the local fine-grained branch *F_local_* and the global sparse large-kernel branch *F_global_*. By concurrently executing global average pooling (GAP) and global maximum pooling (GMP), the module encodes these features through an information bottleneck layer to generate a comprehensive statistical descriptor, *z*. Subsequently, a channel-level Softmax function is employed to dynamically resolve the soft adaptive gating weight vectors *a_c_* and *b_c_* (which strictly adhere to the mathematical constraint *a_c_* + *b_c_* = 1 at all times), thereby enabling the autonomous allocation of the final 3D fused features $\tilde{F_{out}^{\left(i\right)}}$, which is calculated by Equation 2:

(2) $$\tilde{F_{out}^{\left(i\right)}}=a_{c}⊙F_{local}^{\left(i\right)}+b_{c}⊙F_{global}^{\left(i\right)}$$

This mechanism empowers the network with a “flexible” switching capability: it prioritizes local microscopic details when navigating complex intertwined occlusions, while shifting to a large-kernel long-range perspective to infer the holistic plant topology.

Finally, to mitigate the over-smoothing or potential dissipation of high-frequency structural features during Feature Propagation (FP) in deeper layers, we deeply integrated a Deep Large-Kernel Pyramid Pooling Module (DLKPPM) at the terminal stage of the backbone encoder. Diverging from conventional grid-based pooling, the 3D DLKPPM synergizes multi-level Farthest Point Sampling (FPS) with 3D-SDLSKA operators employing stepped dilation rates (e.g., *d=2* and *d=4*). This configuration enables the non-destructive, hierarchical extraction of contextual priors across organs and even canopies—encompassing global statistical states, medium-scale dilated representations, and large-scale, ultra-deep dilated context. Features from various levels are fused via orthogonal concatenation following 3D Inverse Distance Weighted (IDW) interpolation according to Equation 3:

(3) $$F_{DLKPPM}={ℳ}_{fuse}\left(F_{deep}⊕F_{global}⊕F_{mid\_up}⊕F_{large\_up}\right)$$

In summary, the systemic integration of the Large Kernel Attention (LKA) framework does more than just vastly augment the theoretical receptive field threshold of PointNet++ while significantly mitigating memory overflow risks; it further delivers a decisive advantage in point-wise global priors and boundary fidelity for the extraction of 3D crop topologies within the challenging context of salt stress phenotypic monitoring.

#### 2.3.2 DUSAS

Following the implementation of the LKA large-kernel feature aggregation and the BNCV-Loss joint loss function, the Farthest Point Sampling (FPS)—upon which the Set Abstraction (SA) layers of the original PointNet++ architecture heavily rely—exhibits certain geometric and metric limitations. Conventional FPS pursues absolute spatial uniformity based strictly on Euclidean distance; as a result, a substantial portion of the limited sampling budget is inefficiently allocated to information-homogenized flat leaf interiors. In contrast, minute distorted stems or overlapping boundaries, which encapsulate critical high-frequency topological priors, face the risk of being irreversibly discarded. To address these deficiencies, we propose the Dual Uncertainty and Shape-Adaptive Sampling (DUSAS) mechanism. By synergizing local geometric sparsity, latent semantic divergence, and structural curvature saliency within a Riemannian manifold, this mechanism modifies the metric space governing the selection probabilities of candidate points.

Let the input point set of the Set Abstraction (SA) layer be denoted as $V={\left\{v_{j}\right\}}_{j=1}^{N}$, where $v_{j}\in R^{3}$. Let $H={\left\{H_{j}\right\}}_{j=1}^{N}$ represent the set of deep feature vectors mapped through the preceding Multi-Layer Perceptron (MLP), where $\;H_{j}\in R^{D_{h}}$. DUSAS initially defines two independent mathematical uncertainty measures within the continuous geometric space and the high-dimensional feature space, respectively. The first dimension is Geometric Sparsity Uncertainty, which is constructed by retrieving the m nearest neighbor nodes for the candidate point *v_j_* via a K-D tree to form the local support neighborhood $\;\Omega_{m}\left(v_{j}\right)$. Its geometric uncertainty parameter ρ*_j_* is rigorously derived as the second-order moment of the inter-point distance distribution within this neighborhood via Equation 4:

(4) $$\rho_{j}=\frac{1}{\left|\Omega_{m}\left(v_{j}\right)\right|}\sum_{v_{r}\in \Omega_{m}\left(v_{j}\right)}{\left|v_{j}-v_{r}\right|}_{2}^{2}$$

A higher magnitude of ρ*_j_* characterizes a sparser local spatial topology; assigning increased sampling weights to such points serves as a critical physical safeguard for maintaining the 3D topological connectivity of the plant structure. The second dimension is Semantic Divergence Uncertainty. The intensity of fluctuations within the high-dimensional feature space functions as a focal anchor for pinpointing the semantic boundaries of plant organs, such as regions exhibiting stress-induced structural degradation. Semantic divergence, denoted as ζ*_j_*, is quantitatively characterized by the standard deviation of the feature vector across *D_h_* channels. Defining the mean response across the channel dimension as ${\bar{H}}_{j}=\frac{1}{D_{h}}\sum_{w=1}^{D_{h}}H_{j,w}$, the closed-form solution for ζ*_j_* is expressed as follows in Equation 5:

(5) $$\zeta_{j}=\sqrt{\frac{1}{D_{h}}\overset{D_{h}}{\underset{w=1}{\sum }}{\left(H_{j,w}-\bar{H_{j}}\right)}^{2}}$$

To guarantee that the core anchor points strictly adhere to the biological skeletal framework of the plant while filtering out agricultural outlier noise, DUSAS incorporates an adaptive interval partitioning strategy driven by structural saliency. For an arbitrary local neighborhood $\Omega_{m}\left(v_{j}\right)$, a 3D spatial covariance matrix *C_j_* is constructed according to Equation 6, where $\tilde{v_{j}}$ represents the spatial geometric centroid:

(6) $$C_{j}=\frac{1}{\left|\Omega_{m}\left(v_{j}\right)\right|}\underset{v_{r}\in \Omega_{m}\left(v_{j}\right)}{\sum }\left(v_{r}-\tilde{v_{j}}\right){\left(v_{r}-\tilde{v_{j}}\right)}^{T}$$

Performing Singular Value Decomposition (SVD) on the positive semi-definite matrix *C_j_* yields the descending non-negative eigenvalues $E_{1}\geq E_{2}\geq E_{3}\geq 0$, from which the normalized local curvature variation descriptor *k_j_* is derived by Equation 7:

(7) $$k_{j}=\frac{E_{3}}{E_{1}+E_{2}+E_{3}}$$

Subsequently, a lightweight sparse attention mapping operator Γ*_j_*,*_r_* is constructed to compute the implicit semantic affinity between the central point and its neighbors within the local neighborhood. By integrating local curvature saliency, the comprehensive shape-specificity confidence score Γ*_j_*,*_r_* is synthesized for each individual point using Equations 8 and 9:

(8) $$\Gamma_{j,r}=\text{Softmax}\left(\frac{\left(W_{Q}H_{j}\right){\left(W_{K}H_{r}\right)}^{T}}{\sqrt{D_{h}}}\right)$$

(9) $$S_{j}=\underset{v_{r}\in \Omega_{m}\left(v_{j}\right)}{\sum }\Gamma_{j,r}\cdot k_{r}$$

Based on the cumulative distribution function (CDF) of *S_j_*, the system dynamically partitions the 3D point cloud into *B_bins_* probability intervals. Independent dynamic sampling weights, *ϖ_b_*, are then assigned to each interval via self-attention token learning, effectively establishing an optimal trade-off between capturing local structural transitions and maintaining macro-scale global contour uniformity.

During the iterative sampling phase, it is necessary to reconstruct the distance metric penalty function. Min-max normalization is applied to *ρ_j_* and *ζ_j_* within each micro-batch, denoted as $\hat{\rho_{j}}$ and $\hat{\zeta_{j}}$. Let *S* represent the subset of core points already selected at any given step. For the unselected candidate points $v_{j}\notin S$, the selection decision distance Δ*_j_* is reformulated as a nonlinear generalized weighted distance functional that incorporates 4D multimodal information as defined in Equation 10:

(10) $$\Delta_{j}=\varpi_{b\left(j\right)}\cdot \exp\left(\text{η}\cdot \left(\hat{\rho_{j}}+\hat{\zeta_{j}}\right)\right)\cdot \underset{v_{s}\in S}{\min}{\left|v_{j}-v_{s}\right|}_{2}^{2}$$

In this expression, $\varpi_{b\left(j\right)}$ denotes the interval-adaptive weighting constraint, while *η* represents the scale hyperparameter responsible for modulating the exponential amplification of sensitivity toward dual uncertainties. The iterative logic strictly adheres to a greedy selection strategy, wherein $v^{*}=\arg\underset{v_{j}\notin S}{\max}\Delta_{j}$ is determined at each step to identify the optimal candidate. The efficacy of this mathematical framework resides in its adaptive capacity: even for candidate points obscured within dense foliar interiors, the nonlinear exponential amplifier exp(·) triggers a substantial gain to forcibly elevate their selection probability, provided they reside within morphological distortion zones or exhibit extreme latent feature instability (characterized by maximized $\hat{\rho_{j}}$ or $\hat{\zeta_{j}}$).

The DUSAS mechanism effectively upgrades primitive static geometric distance searches into a paradigm that integrates deep features and spatial uncertainty to guide the sampling process. This uncertainty-aware sampling paradigm effectively mitigates the long-tail effects associated with the loss of critical edge points during point cloud downsampling, thereby providing a high-density foundational skeletal structure for subsequent global attention modules and boundary polarization losses.

#### 2.3.3 BNCV-loss

Despite the success of the aforementioned Large Kernel Attention (LKA) mechanism in bypassing local receptive field constraints and securing long-range global contextual dependencies, it remains highly susceptible to the “feature smoothing” effect during deep feature mapping. This susceptibility often results in the assimilation of high-frequency geometric details—particularly at the critical transitions between the primary stem, petioles, and cotyledons—by the overwhelming abundance of low-frequency internal point features. Furthermore, the morphological distortions exhibited by seedlings under salt stress, characterized by extremely thin withered stems and clustered, overlapping broad leaves, manifest as an exceptionally extreme long-tail data distribution and scale imbalance within the 3D spatial domain. To seamlessly bridge the fine-grained boundary deficiencies of LKA and address extreme inter-class imbalance, we formulate the Boundary-Aware Nested Contrastive and Adaptive Varifocal Joint Loss (BNCV-Loss)—a joint loss function that combines three distinct sub-modules: Nested Contrastive Boundary Loss (NCBL), 3D Adaptive Varifocal Loss (3D-VFL), and a Geometric Structural Similarity (GASS) prior. During error backpropagation, this coordinated mechanism yields high-frequency compensatory gradients that operate in direct opposition to the spatial low-pass smoothing effect induced by LKA.

Concurrently, to mitigate the dominance of background tokens, a nonlinear decay factor $-\alpha p_{i}^{\gamma }$ rapidly attenuates redundant gradients from the overwhelming majority of easily classified samples (*y_i_* = 0) to an infinitesimal level, thereby enabling the model to detect millimeter-scale fine features. Furthermore, across smooth organ surfaces, the GASS prior enforces a stringent structural penalty, compelling the decoder output to maintain structural and spatial consistency across smooth organ surfaces.

In scenarios characterized by complex overlaps and curled morphologies, traditional Euclidean distance is prone to inducing misjudgments of local neighborhood features. Consequently, following the FPS stage, we incorporate geodesic distance *D_geo_*(*x_i_*, *x_k_*) based on local graph connectivity to extract the authentic anatomical semantic boundary point set $B_{l}=\left\{x_{i}\in X|\exists x_{k}\in N_{i}^{\text{geo}}\wedge l_{i}\neq l_{k}\right\}$. For any boundary centroid $x_{i}\in B_{l}$ situated at a semantic interface, its geodesic neighborhood $N_{i}^{\text{geo}}$ is strictly and mutually exclusively partitioned into intra-class boundaries *P*_1_, intra-class interior regions *P*_2_ and inter-class neighborhoods. To forestall the irreversible collapse of boundary features toward the characteristics of the interior hinterland (Kervadec et al., 2019), the *NCBL* introduces an adaptive nested weight ${\text{ω}}_{in}\in \left(0,1\right)$, leading to the derivation of the nested contrastive objective function in Equation 11:

(11) $${ℒ}_{NCℬℒ}=-\frac{1}{\left|B_{l}\right|}\underset{x_{i}\in B_{l}}{\sum }\log\left(\frac{\sum_{x_{k}\in P_{1}}\exp\left(-\frac{d\left(f_{i},f_{k}\right)}{\tau }\right)+\omega_{in}\sum_{x_{k}\in P_{2}}\exp\left(-\frac{d\left(f_{i},f_{k}\right)}{\tau }\right)}{\sum_{x_{k}\in N_{i}^{geo}}\exp\left(-\frac{d\left(f_{i},f_{k}\right)}{\tau }\right)}\right)$$

where *d*(*f_i_*, *f_k_*) represents a nonlinear mapping of the cosine distance between feature vectors, and *τ* is a temperature scaling hyperparameter. This compels the LKA to autonomously manifest a sharp feature distinction at physical boundaries, facilitating the high-precision delineation and separation of adhered and overlapping leaves.

To address the challenge where minute stems and newborn cotyledons constitute an extremely low proportion of the point cloud—leading to a pronounced deficit in recall performance due to the dominance of backgrounds or large mature leaves—we introduce a continuous supervisory signal *q_i_* based on 3D Intersection over Union (3D-IoU) perception. For the classification prediction probability *p_i_* of a discrete point *i*, the 3D-VFL disrupts the symmetry of positive and negative sample weights, restructuring the supervisory logic through a piecewise differential equation shown in Equation 12:

(12) $${ℒ}_{VFL}\left(p_{i},q_{i}\right)=\{\begin{array}{ll} -q_{i}\left(q_{i}\log\left(p_{i}\right)+\left(1-q_{i}\right)\log\left(1-p_{i}\right)\right), & ify_{i}>0\\ -\alpha p_{i}^{\gamma }log\left(1-p_{i}\right), & ify_{i}=0 \end{array}$$

When a data point belongs to a minority critical organ such as a fragile stem (*y_i_* > 0), the coefficient *q_i_* guides the network not only toward correct classification but also toward the joint alignment of the probability confidence *p_i_* with the ground-truth spatial fidelity *q_i_*.

Reliance solely on point-level feature space loss often leads to local fragmented noise or geometric tearing in the network’s predictive output. To ensure that 3D segmentation clusters strictly adhere to the physical coherence of the plant, we extract the local surface normal field $\nabla n_{m}$ and the discrete principal curvature $\nabla k_{m}$ as foundational physical prior descriptors. By combining these with local statistics of the network-predicted features and ground-truth labels (mean *μ*, variance σ^2^ and covariance σ*_pg_*), the following 3D Structural Similarity Loss term is constructed as Equation 13:

(13) $${ℒ}_{\text{GASS}}=1-\frac{1}{\left|M\right|}\underset{m\in M}{\sum }\left[\left(\frac{2{\text{μ}}_{p}{\text{μ}}_{g}+c_{1}}{{\text{μ}}_{p}^{2}+{\text{μ}}_{g}^{2}+c_{1}}\right)\left(\frac{2{\text{σ}}_{pg}+c_{2}}{{\text{σ}}_{p}^{2}+{\text{σ}}_{g}^{2}+c_{2}}\right)\cdot \Psi \left(\nabla n_{m},\nabla k_{m}\right)\right]$$

In this expression, $\Psi \left(\nabla n_{m},\nabla k_{m}\right)$ serves as a curvature-aware gating operator based on a Gaussian kernel distribution. When the local mesh calculation detects natural mutations in surface geometry (such as leaf vein ridges), the operator relaxes the smoothing constraints; within smooth organ surfaces, however, it imposes a stringent penalty, thereby enabling the decoder to maintain structural and spatial consistency across smooth organ surfaces.

Ultimately, the BNCV-Loss joint optimization objective function is formulated as a multi-term non-convex joint loss optimization function in Equation 14:

(14) $${ℒ}_{total}={ℒ}_{VFL\_total}+\lambda_{1}{ℒ}_{NCBL}+\lambda_{2}{ℒ}_{GASS}+\beta {\left\|\Theta \right\|}_{2}^{2}$$

where λ_1_ and λ_1_ are balancing hyperparameters that adaptively evolve based on task-variance uncertainty weighting. The term $\text{β}{\left\|\text{Θ}\right\|}_{2}^{2}$ represents the norm weight decay. The physical integration of these three gradient vectors within the backpropagation channel effectively addresses the high-frequency feature attenuation defects of the LKA architecture, improving plant point cloud organ segmentation at the sub-millimeter level.

#### 2.3.4 Calculation of broccoli seedling phenotypic traits

Following the semantic and instance segmentation of broccoli seedling organs, this study utilizes a suite of mathematical models based on point cloud geometric and topological properties to achieve automated quantitative extraction of key phenotypic parameters (Qiu et al., 2025). To establish a unified baseline for height measurement, the algorithm first employs the Random Sample Consensus (RANSAC) algorithm to geometrically model the substrate plane of the cultivation environment. A ground reference plane equation is fitted via robust linear regression as Equation 15:

(15) z=ax+by+d

This equation establishes a 3D reference coordinate system for plant growth, where (*x*, *y*) represents the horizontal spatial coordinates and z denotes the vertical height information. Subsequently, the system applies the Density-Based Spatial Clustering of Applications with Noise (DBSCAN) algorithm to perform individual clustering on the decoupled plant point clouds (Lei et al., 2025). By setting a core neighborhood radius of ε=2.5cm and a minimum point threshold of minPts = 20, the point cloud is partitioned into independent physical observation units, laying the foundation for subsequent individual-plant phenotypic analysis.

Regarding the calculation of plant height (H), to eliminate interference from extreme outliers potentially generated during the 3D reconstruction process and to enhance measurement robustness, the algorithm extracts the statistical distribution characteristics of the individual plant point cloud along the vertical axis. The absolute height value of the plant is defined as the difference between the 99 percentile height and the 1 percentile height according to Equation 16:

(16) $$H=Z_{99\%}-Z_{1\%}$$

This method accurately reflects the growth retardation of broccoli seedlings under salt stress (Zhao et al., 2025). To characterize the light interception capacity and biomass accumulation levels, the projected leaf area (A) and seedling volume (V) are solved using Convex Hull geometric theory (Zhao et al., 2018). The projected leaf area is obtained by calculating the minimum convex hull area of the individual plant point cloud on the horizontal projection plane, expressed mathematically in Equation 17:

(17) $$A=\iint_{CH_{2}D}dx\;dy$$

where *CH*_2_*D* represents the 2D convex hull of the point set on the *xy* plane. Seedling volume is realized by constructing the minimum 3D convex polyhedron that encloses the individual plant entity. The volume index *V* is defined as the spatial capacity occupied by this 3D convex hull via Equation 18:

(18) $$V={∭}_{CH_{3}D}dx\;dy\;dz$$

Through the aforementioned algorithmic workflow, the system can precisely capture subtle phenotypic variations—such as reduced plant height, diminished projected leaf area, and inhibited biomass accumulation—in broccoli seedlings across various salt stress gradients, providing a high-throughput digital evaluation foundation for salt-tolerant broccoli breeding.

#### 2.3.5 Architectural and hyperparameter details of LBD-PointNet++

To ensure complete transparency and algorithmic reproducibility for researchers, the specific engineering structural configurations and hyperparameters for all proposed modules within LBD-PointNet++ are comprehensively consolidated in **Table 1**. These parameters were determined via systematic grid searching on a 10% validation subset to balance memory consumption and computational throughput.

**Table 1:** Detailed architectural and training hyperparameters for reproducibility.

Module	Structural component/hyperparameter	Operational setting/value	Selection justification/protocol
LKA Block	Large Separable Kernels (1×1×K, 1×K×1)	*K* = 7	Captures multi-row local dependencies on tray manifold
	Axial Stepped Dilation Strides (d)	d∈{1,2,4}	Decouples linear complexity while extending field range
	Feature Dimensions across SA Layers	[64, 128, 256, 512]	Standard hierarchical scale-up for channel capacity
DUSAS	Local Neighbor Window (M via K-D Tree)	*M* = 20 *points*	Optimizes local curvature estimation and saliency maps
	Softmax Token Learning Head Count	4 heads	Captures multi-dimensional affinity in latent feature space
	Saliency Adaptive Partitioning Intervals	B_bins_ = 4	Optimal trade-off between flat interior and micro-stems
	Exponential Sensitivity Modulation Scale	η = 0.5	Modulates non-linear acceleration of dual uncertainty
BNCV-Loss	Nested Weight for Interior hinterland	ω_in_ = 0.35	creates a steep sharp feature polarization to separate overlapping leaves
	Temperature Scaling Parameter	τ = 0.07	Standard contrastive alignment scaling factor
	Loss Balancing Trade-offs (λ_1_, λ_2_)	λ_1_ = 1.0 λ_2_ = 0.5	Balances Varifocal coverage with contrastive boundaries
	Norm Weight Decay Coefficient	β = 1×10^−4^	Standard L_2_ regularization penalty for overfitting

#### 2.3.6 Model evaluation metrics

To scientifically and comprehensively evaluate the organ-level semantic segmentation performance of the improved LBD-PointNet++ model in salt-stressed environments, we selected Intersection over Union (IoU), Precision, Recall, and the F1-score as our core evaluation metrics. Considering broccoli’s status as a fundamental vegetable rich in bioactive compounds, it exhibits significant phenotypic abnormalities under salt stress, such as stunted height and compressed 3D volumetric expansion. Traditional segmentation models often struggle to capture these subtle geometric changes. Therefore, a comprehensive evaluation through these four metrics not only validates the model’s recognition accuracy across “Leaf,” “Stem,” and “Pot” semantic categories but also systematically quantifies the superiority of the LKA mechanism in capturing long-range dependencies, the BNCV-Loss in enhancing boundary precision, and the DUSAS mechanism in preserving high-frequency topological features.

In the specific mathematical definitions and statistical processes, we utilize True Positives (TP), False Positives (FP), and False Negatives (FN) as the fundamental computing units. TP represents the number of points correctly predicted by the model as the target category; FP denotes the number of non-target category points incorrectly predicted as that category; and FN indicates the number of target category points that were missed. Precision measures the purity of the prediction results, reflecting the proportion of points truly belonging to a specific organ among all points identified as that organ. Recall emphasizes the model’s coverage capability, demonstrating its sensitivity in capturing small-scale features when faced with morphological distortions induced by salt stress. To establish an optimal trade-off between precision and recall and effectively address the challenge of extreme imbalance in plant organ point cloud distribution, we introduced the F1-score as their harmonic mean. Additionally, IoU serves as the most critical metric for evaluating 3D semantic segmentation quality; by calculating the degree of overlap between the predicted point set and the ground-truth labels, it visually demonstrates the reconstruction accuracy of the LBD-PointNet++ model for the plant’s 3D topological structure. Specifically, it highlights the boundary stripping effect produced by the sharp feature polarization of the BNCV-Loss on adhered and overlapping leaf margins. The specific calculation formulas for each metric are as follows in Equations 19 to 22:

(19) $$Precision=\frac{TP}{TP+FP}$$

(20) $$Recall=\frac{TP}{TP+FN}$$

(21) $$F1-score=2\times \frac{Precision\times Recall}{Precision+Recall}$$

(22) $$IoU=\frac{TP}{TP+FP+FN}$$

## 3 Results

### 3.1 Operating environment

To evaluate the effectiveness of the proposed improved PointNet++ model in the phenotypic analysis of broccoli seedlings, all algorithms were trained and tested on a workstation equipped with a high-performance Graphics Processing Unit (GPU). As detailed in **Table 2**, the system is configured with a processor featuring 16 logical threads and 16 GB of system RAM, providing sufficient computational capacity for the preprocessing of large-scale point cloud data. The core computational tasks are performed by an NVIDIA GeForce RTX 4050 Laptop GPU with 6 GB of dedicated video memory, which supports CUDA 12.4 acceleration. This hardware configuration enables the efficient handling of high-dimensional tensor computations involved in 3D spatial convolutions and large-kernel attention mechanisms. On the software side, the experimental environment was hosted on the Windows 11 operating system, with core algorithms developed in Python 3.13 using the PyTorch 2.6.0 deep learning framework to implement the model’s forward inference and backpropagation.

**Table 2:** Experimental system configuration.

Category	Specifications
OS	Windows 11
CPU	16-core Logic Processor
GPU	NVIDIA RTX 4050 (6GB)
RAM	16 GB
Python Version	3.13.0
CUDA Version	12.4
Framework	PyTorch 2.6.0

In the specific model training phase, a suite of critical hyperparameters was established based on the morphological characteristics of broccoli seedlings to achieve an optimal balance between generalization performance and convergence speed. As detailed in **Table 3**, the training procedure encompassed 50 iterations (Epochs), employing a Batch Size of 16. Each input point cloud was normalized to 4,096 feature points (Npoint) via a sampling algorithm. To facilitate rapid convergence toward the global optimum during initial training and to suppress late-stage oscillations, the Adam optimizer was utilized with a starting Learning Rate of 0.001. Additionally, a learning rate scheduling mechanism was implemented, whereby the learning rate was adjusted by a Decay Rate of 0.7 every 10 Epochs. To minimize the risk of overfitting, the Weight Decay coefficient was strictly maintained at 0.0001. Such rigorous environmental configurations and parameterizations ensure the model’s precision in segmenting millimeter-scale anatomical components, including stems, leaves, and cultivation containers.

**Table 3:** Hyperparameters for model training.

Parameter	Specifications
Total Epochs	50
Batch Size	16
Initial Learning Rate	0.001
Optimizer	Adam
Npoint	4096
Learning Rate Decay Step	10
Learning Rate Decay Rate	0.7
Weight Decay	0.0001

### 3.2 Ablation experiments

To validate the effectiveness of the proposed Large Kernel Attention (LKA) mechanism, Boundary-Aware Nested Contrastive and Adaptive Varifocal Joint Loss (BNCV-Loss), and Dual Uncertainty and Shape-Adaptive Sampling (DUSAS) in the organ segmentation task of broccoli seedlings, a series of systematic ablation experiments were conducted using the control variable method. With the original PointNet++ serving as the baseline model, the experimental results are summarized in **Table 4**; each improvement strategy contributed positively to the model’s overall performance. First, the introduction of the LKA module significantly enhanced the model’s capacity to capture global plant topology and long-range dependencies, increasing the mIoU from 84.62% to 85.15%, while the Mean Precision (mP) rose markedly from 90.25% to 92.10%. Subsequently, the independent integration of BNCV-Loss yielded a distinct advantage in Mean Recall (mR), which reached 90.30%; this provides strong evidence of the loss function’s superior performance in addressing class imbalance between fragile stems and backgrounds under salt stress, as well as in delineating fine edge details. Furthermore, the DUSAS mechanism, through its dual uncertainty-driven sampling strategy, effectively preserved key points containing high-frequency topological information. By preventing the loss of minute organ features during the downsampling process, it achieved an mIoU of 85.54%.

**Table 4:** Ablation study of the proposed modules on organ-level semantic segmentation performance for broccoli seedlings.

Index	Part	IoU	Precision	Recall	F1-score
Pointnet++	Pot	96.07	97.53	98.47	98.00
	Leaf	83.07	91.48	90.04	90.75
	Stem	73.85	86.52	83.45	84.96
	Mean	84.33 ± 0.68	91.84 ± 0.62	90.65 ± 0.75	91.24 ± 0.70
+LKA	Pot	96.29	97.61	98.62	98.11
	Leaf	83.92	92.05	90.48	91.26
	Stem	74.95	87.18	84.23	85.68
	Mean	85.05 ± 0.60	92.28 ± 0.55	91.11 ± 0.68	91.68 ± 0.58
+BNCV-Loss	Pot	96.48	97.74	98.68	98.21
	Leaf	84.36	92.23	90.81	91.51
	Stem	75.38	87.45	84.52	85.96
	Mean	85.41 ± 0.63	92.47 ± 0.52	91.34 ± 0.59	91.89 ± 0.54
+DUSAS	Pot	96.60	97.82	98.73	98.27
	Leaf	84.67	92.47	90.94	91.70
	Stem	75.74	87.76	84.68	86.19
	Mean	85.67 ± 0.58	92.68 ± 0.57	91.45 ± 0.64	92.05 ± 0.56
+LKA +BNCV-Loss	Pot	96.78	97.85	98.88	98.36
	Leaf	85.50	92.96	91.42	92.18
	Stem	76.33	88.15	85.06	86.58
	Mean	86.20 ± 0.54	92.99 ± 0.50	91.79 ± 0.56	92.37 ± 0.49
+LKA +DUSAS	Pot	96.93	97.93	98.96	98.44
	Leaf	86.15	93.38	91.75	92.56
	Stem	77.11	88.67	85.54	87.08
	Mean	86.73 ± 0.55	93.33 ± 0.46	92.08 ± 0.52	92.69 ± 0.45
+BNCV-Loss +DUSAS	Pot	97.19	98.04	99.12	98.58
	Leaf	86.84	93.75	92.18	92.96
	Stem	77.54	88.93	85.82	87.35
	Mean	87.19 ± 0.52	93.57 ± 0.49	92.37 ± 0.46	92.96 ± 0.42
LBD-PointNet++	Pot	97.52	98.22	99.27	98.74
	Leaf	88.00	94.46	92.79	93.62
	Stem	78.70	89.78	86.45	88.08
	Mean	88.07 ± 0.51	94.15 ± 0.38	92.84 ± 0.42	93.48 ± 0.35

In terms of the synergistic effects of module combinations, the progressive superposition of different modules exhibits a mathematically sound and saturating trend under the disjoint protocol. The integrated LBD-PointNet++ framework yields a cohesive performance gain due to the multi-level physical synergy of the three modules: DUSAS acts as a structural gatekeeper that preserves fine geometric boundaries, supplying the critical morphological anchors that the LKA block requires to capture long-range global dependencies without voxelized collapse. Concurrently, BNCV-Loss enforces high-frequency gradient compensation during training that directly counters the feature-smoothing or low-pass blurring effect inherent to deep large-kernel attention backbones. This closed-loop optimization across the data sampling, architectural representation, and gradient backpropagation levels explains the robust global accuracy. To ensure the statistical reliability of these performance metrics given the limited sample size, and to mitigate the risk of overfitting to a specific data split, we conducted a 5-fold cross-validation using three different random initialization seeds. Ultimately, the fully integrated LBD-PointNet++ model demonstrated robust stability, achieving an average mIoU of 88.07% (± 0.51%) across all three categories (Leaf, Stem, and Pot) and an average F1-score of 93.48% (± 0.35%). These results fully demonstrate the robust performance of the deep coupling between LKA, BNCV-Loss, and DUSAS in handling morphological distortions and complex overlapping scenarios of broccoli seedlings, successfully demonstrating exceptional capability in preserving fine-grained structural details under complex stress conditions.

Beyond the aforementioned segmentation metrics, we further evaluated the model’s equilibrium between computational complexity and Overall Accuracy (OA). As detailed in **Table 5**, the baseline PointNet++ model maintained a streamlined profile with a parameter count of 0.8510 M and a memory footprint of 122.40 MB, while achieving an OA of 93.85%. Upon the introduction of the LKA module, the parameter count increased slightly to 0.9670 M, yet the OA significantly ascended to 94.90%. This demonstrates that the large-kernel attention mechanism, leveraging its 3D sparse decomposition strategy, secures substantial recognition gains with only a marginal consumption of additional computational resources. The integration of BNCV-Loss extended the running time to 385.12 ms, primarily due to the intensive high-frequency gradient compensation calculations involved in the nested contrastive boundary loss during backpropagation; nevertheless, this module successfully optimized the OA to 94.65%. Furthermore, the incorporation of the DUSAS mechanism raised the parameter count to 1.0820 M—attributed to the execution of dual uncertainty measures within continuous geometric and high-dimensional feature spaces—consequently enhancing the OA to 94.82%.

**Table 5:** Trade-off analysis between computational efficiency and overall accuracy for modular configurations of LBD-PointNet++.

Index	Params (M)	Mem (MB)	Time (ms)	OA
Pointnet++	0.8510	122.40	335.20	93.85
+LKA	0.9670	135.18	421.43	94.90
+BNCV-Loss	0.8510	122.40	335.20	94.65
+DUSAS	1.0820	151.13	475.65	94.82
+LKA +BNCV-Loss	0.9670	158.69	491.96	96.10
+LKA +DUSAS	1.0980	173.03	527.95	96.65
+BNCV-Loss +DUSAS	1.0820	161.54	569.01	96.30
LBD-PointNet++	1.0980	185.42	610.12	97.35

During the deep coupling of multiple modules, although the computational overhead accumulated alongside functionality enhancements, the precision gains exhibited a superior cost-performance ratio. The finally proposed LBD-PointNet++ model attained a high overall mIoU of 88.07% across all three categories (Leaf, Stem, and Pot), representing a significant, unbiased representation gain on biological organs. While the inference latency for the complete model increased to 610.12 ms, this significant leap in accuracy is vital for managing complex scenarios where broccoli seedlings exhibit extreme morphological distortion under salt stress. By substantially reducing manual verification costs, these results fully validate the engineering application value and algorithmic robustness of LBD-PointNet++ for practical high-throughput phenotypic monitoring tasks.

### 3.3 Comparative performance analysis across different segmentation models

**Figure 5:**
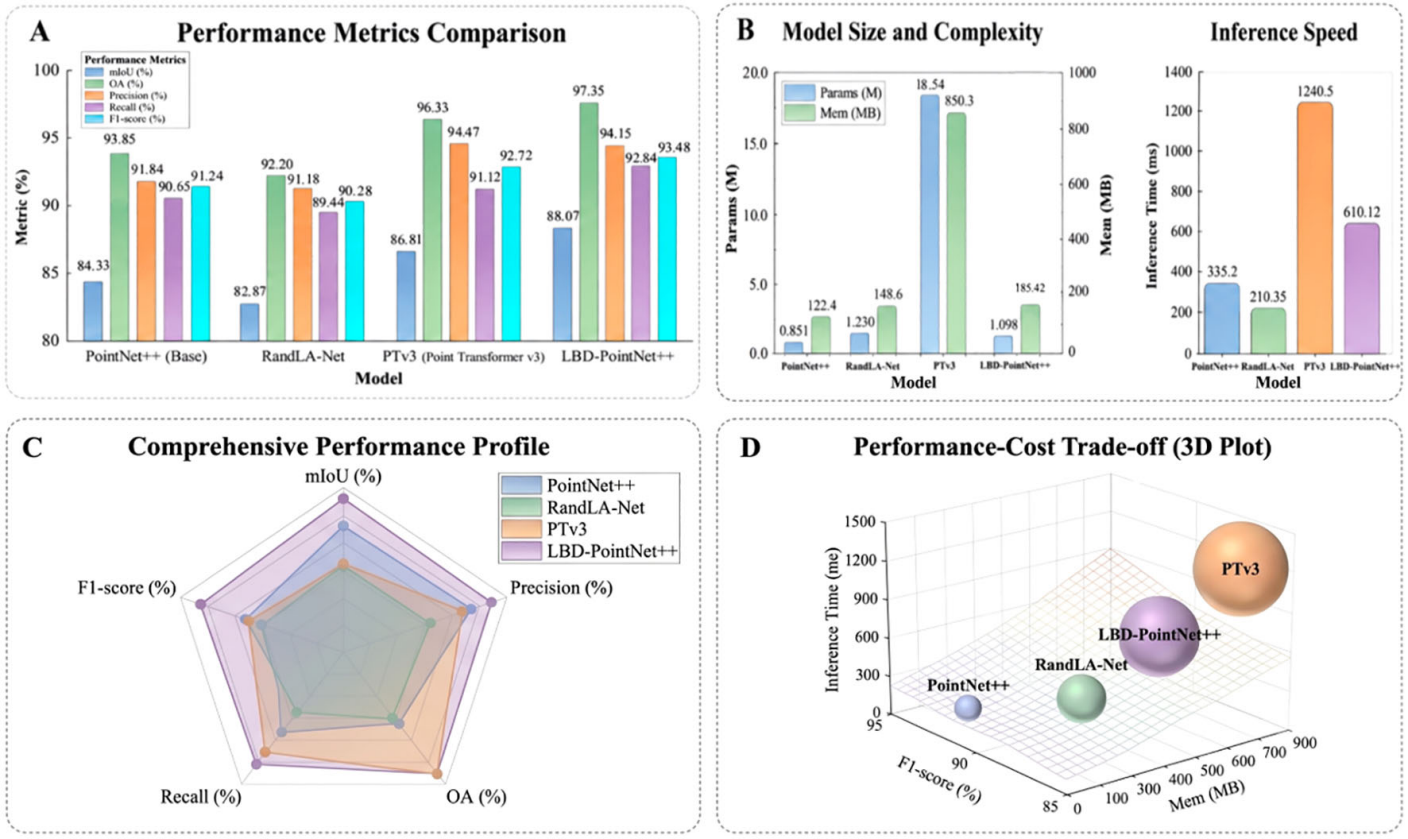
**(A)** Comparison of core segmentation performance metrics. **(B)** Quantitative evaluation of model complexity (parameters) versus inference latency. **(C)** Radar chart visualization for holistic performance assessment across multiple evaluation dimensions. **(D)** 3D scatter plot of the trade-off between segmentation accuracy, computational cost, and memory footprint.

To substantiate the superior performance of LBD-PointNet++ in the phenotypic analysis of broccoli seedlings under salt stress, we conducted a rigorous and impartial benchmarking against the baseline PointNet++, the efficiency-oriented RandLA-Net, and the state-of-the-art Transformer-based architecture, PTv3 (Wu et al., 2024). To guarantee an absolute and transparent baseline alignment, all models were trained and tested on the identical broccoli seedling dataset under a standardized training protocol: utilizing a unified input point scale of N = 4,096, the same data augmentation routines, the Adam optimizer with an identical learning rate schedule (initial LR of 0.001, decaying by 0.7 every 10 epochs), a fixed batch size of 16, and an identical total duration of 50 epochs. Crucially, since PTv3 was originally engineered for million-point indoor/outdoor scenes, directly running its default script on sparse 4,096-point inputs would cause severe representational collapse and an unfair efficiency deficit. Therefore, PTv3 was fine-tuned leveraging weights pre-trained on the ScanNet dataset. Furthermore, its initial voxel grid downsampling dimension was significantly downscaled and its internal spherical neighbor searching radius was tightly shrunk to align with the sub-centimeter inter-plant spaces of our culture trays. This tailor-made grid tuning ensures that PTv3 operates within its optimal capacity range on our data, verifying the scientific integrity of the comparative results reported in **Figure 5**.

Regarding architectural performance, RandLA-Net (Hu et al., 2020), which utilizes efficient random sampling to process large-scale point clouds, exhibits a notable inference speed advantage, with a latency of only 210.35 ms. However, as it is primarily designed for large-scale scenes, its stochastic sampling mechanism—while significantly boosting processing efficiency—is prone to overlooking the minute geometric features induced by salt stress. Consequently, its mIoU of only 82.87% fails to meet the stringent requirements for sub-millimeter phenotypic extraction. PointNet++, a classic hierarchical feature learning framework, demonstrates competent local shape capture with the lowest memory footprint in the study (122.4 MB). Nevertheless, its reliance on a fixed-radius ball query mechanism limits its precision when navigating complex topological distortions, such as leaf curling and stem atrophy. In these instances, single-scale local modeling frequently induces boundary smoothing effects, thereby constraining segmentation accuracy. As a leading Transformer-based architecture, PTv3 achieves a high precision of 91.04% on stems by leveraging serialized neighborhood mapping and an ultra-large receptive field. However, it must be emphasized that PTv3 was originally engineered to possess massive representation capacity for multi-million-point indoor/outdoor scenes. When forced into an ultra-sparse regime of 4,096 points per individual plant patch, the heavy voxel-gating and serialized cross-attention paths of PTv3 operate far outside their intended architectural design, leading to disproportionate latency (1240.5 ms) and memory bloat. Therefore, this benchmarking should not be interpreted as a direct like-for-like comparison but rather demonstrates that our network is specifically optimized as a lightweight alternative for resource-constrained sparse agricultural phenotyping backends.

In contrast, LBD-PointNet++ demonstrates a distinct competitive edge in this performance benchmarking. It not only secures the highest scores across core accuracy metrics—achieving an mIoU of 88.07% across all three categories (Leaf, Stem, and Pot), mP of 94.15%, mR of 92.84%, and an F1-score of 93.48%—but also provides a substantial efficiency advantage over PTv3. The data reveals a pivotal fact: LBD-PointNet++ surpasses PTv3 in segmentation accuracy while utilizing less than 6% of its parameter count and more than doubling the inference speed. This exceptional performance is primarily attributed to the LKA large-kernel attention mechanism’s acute perception of global context and the DUSAS sampling mechanism’s precise preservation of high-frequency topological features. Particularly when resolving heavily overlapped leaf margins and fragile stressed stems, LBD-PointNet++ achieves sub-millimeter precision in decoupling and segmentation. It effectively bridges the long-standing contradiction between feature smoothing and computational redundancy, offering the current optimal balance of lightweight design and high-precision phenotyping for Brassicaceae crops under salt stress.

**Figure 6:**
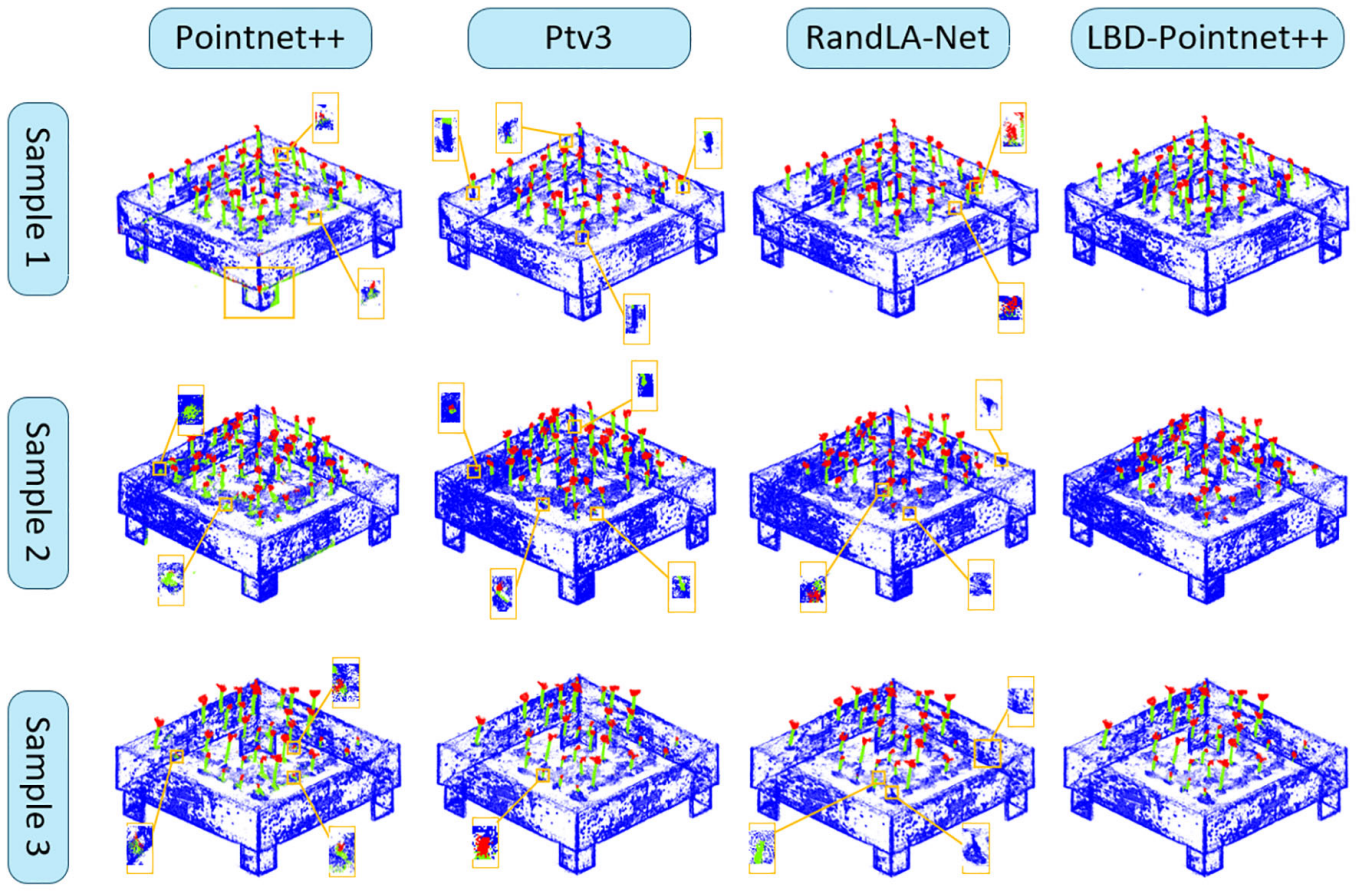
Qualitative visual comparative analysis of semantic segmentation results on broccoli seedling point clouds across different models.

**Figure 6** illustrates the comparative visual segmentation results of PointNet++, PTv3, RandLA-Net, and our proposed LBD-PointNet++ across three representative specimens (Sample 1, 2, and 3). In the visualization, red, green, and blue denote leaves, stems, and cultivation containers, respectively, while yellow bounding boxes highlight magnified views of critical local segmentation regions.

In Sample 1, which features a relatively regular plant distribution, performance disparities among the models are already evident. Due to its stochastic sampling mechanism, RandLA-Net suffers from significant point cloud feature depletion, resulting in a visually fragmented skeleton for the fragile stems. In contrast, while PointNet++ successfully recovers the general plant contour, it exhibits localized semantic ambiguity at the leaf-stem junctions near the base. PTv3, leveraging the global perception capabilities of its Transformer architecture, achieves a competent overall segmentation but lacks sufficient noise suppression along the edges of the background container. This superiority is largely attributed to their self-attention mechanisms. For instance, the Point Cloud Transformer (PCT) introduces an offset-attention mechanism that refines features by calculating the offset between input and attention features, proving highly effective for point cloud learning (Guo et al., 2021).

As structural density increases in Sample 2, the models’ capacity to handle complex topological features is further tested. PointNet++, constrained by its fixed-radius ball query mechanism, exhibits a pronounced “feature smoothing” effect when faced with overlapping leaves and intertwined stems, leading to the misclassification of certain stem segments as leaves. While PTv3 maintains high precision in this dense scenario, the magnified regions still reveal minor “leakage,” where red leaf points penetrate the green stem zones. At this stage, RandLA-Net struggles to maintain even the most basic plant anatomical topology.

In Sample 3, which is characterized by extreme morphological distortions under salt stress, the robustness of LBD-PointNet++ is most intuitively demonstrated. Conventional PointNet++ suffers from massive stem fractures and fails to capture the subtle features of withered, thin stems. Even within PTv3, edge classification errors caused by inter-class imbalance are observable in the detailed magnifications. Conversely, LBD-PointNet++ exhibits marked superiority across all samples. It not only accurately extracts millimeter-scale stressed stems in Sample 3 but also effectively resolves the adhesion of overlapping leaf margins through the sharp feature polarization effect induced by BNCV-Loss. These visual results provide direct evidence that LBD-PointNet++ possesses optimal topological maintenance and boundary segmentation precision when confronted with complex phenotypes and distorted morphologies.

### 3.4 Analysis of broccoli seedling phenotypic responses under different concentrations of NaCl stress

The ultimate objective of proposing LBD-PointNet++ is to overcome the severe physical and geometric limitations of 2D pixel-level imaging in high-throughput crop phenotyping. Conventional 2D semantic and instance segmentation architectures utilizing the RGB images captured by our system suffer from irreversible perspective occlusion, view-angle dependency, and a fundamental lack of spatial depth information. Consequently, they are inherently incapable of untangling multi-layered leaf overlaps or computing the true 3D spatial volumetric occupancy of curled, salt-stressed leaves. By leveraging the superior high-fidelity 3D segmentation masks generated by LBD-PointNet++, the phenotyping pipeline can seamlessly transition to precise, non-destructive 3D trait extraction. To rigorously justify the necessity of 3D point cloud analysis, we benchmarked our framework against three industry-standard conventional 2D segmentation models: Mask R-CNN (He et al., 2017), YOLOv8-seg (Jocher et al., 2023), and DeepLabv3+ (Chen et al., 2018). The quantitative segmentation performance comparison is comprehensively summarized in **Table 6**.

**Table 6:** Quantitative segmentation performance comparison between conventional 2D image-based models and the proposed 3D point cloud network under 5-fold cross-validation.

Index	MioU*	MP	MR	F1-score
Mask R-CNN	73.25 ± 0.72	84.12 ± 0.65	76.80 ± 0.81	80.29 ± 0.68
YOLOv8-seg	75.68 ± 0.68	86.45 ± 0.58	78.92 ± 0.74	82.51 ± 0.61
DeepLabv3+	74.15 ± 0.70	85.20 ± 0.61	77.45 ± 0.79	81.14 ± 0.64
LBD-PointNet++	88.07 ± 0.51	94.15 ± 0.38	92.84 ± 0.42	93.48 ± 0.35

(The ‘Mean’ IoU is calculated as the average of the IoU values for all three categories: leaf, stem, and pot).

**Figure 7:**
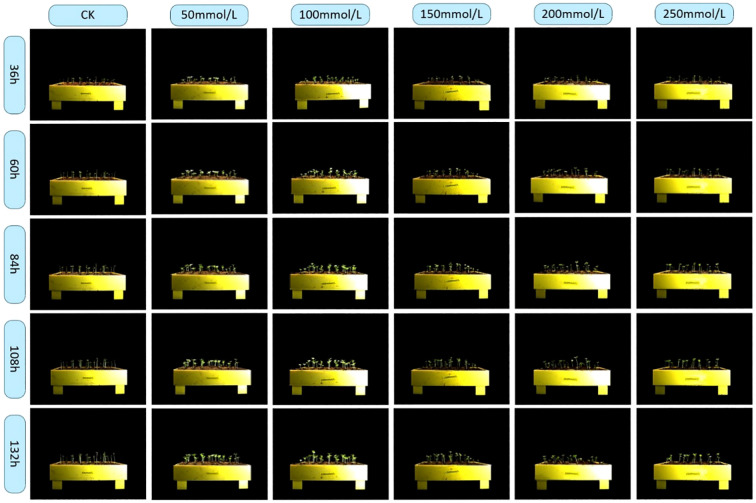
Front-view sequences of broccoli seedling growth under various NaCl concentration treatments.

**Figure 8:**
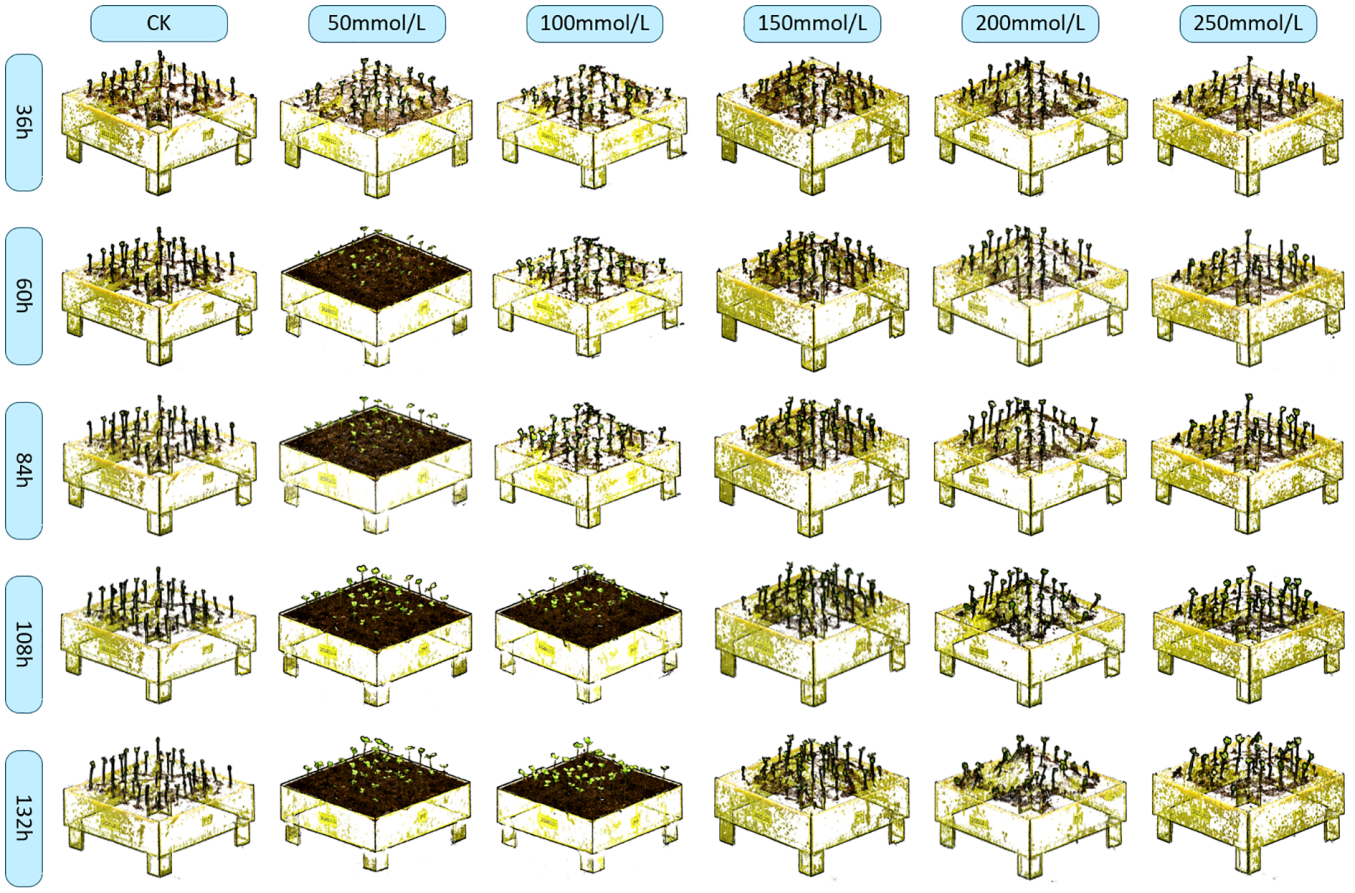
3D point cloud reconstruction results of broccoli seedlings under various NaCl concentration treatments.

**Figure 9:**
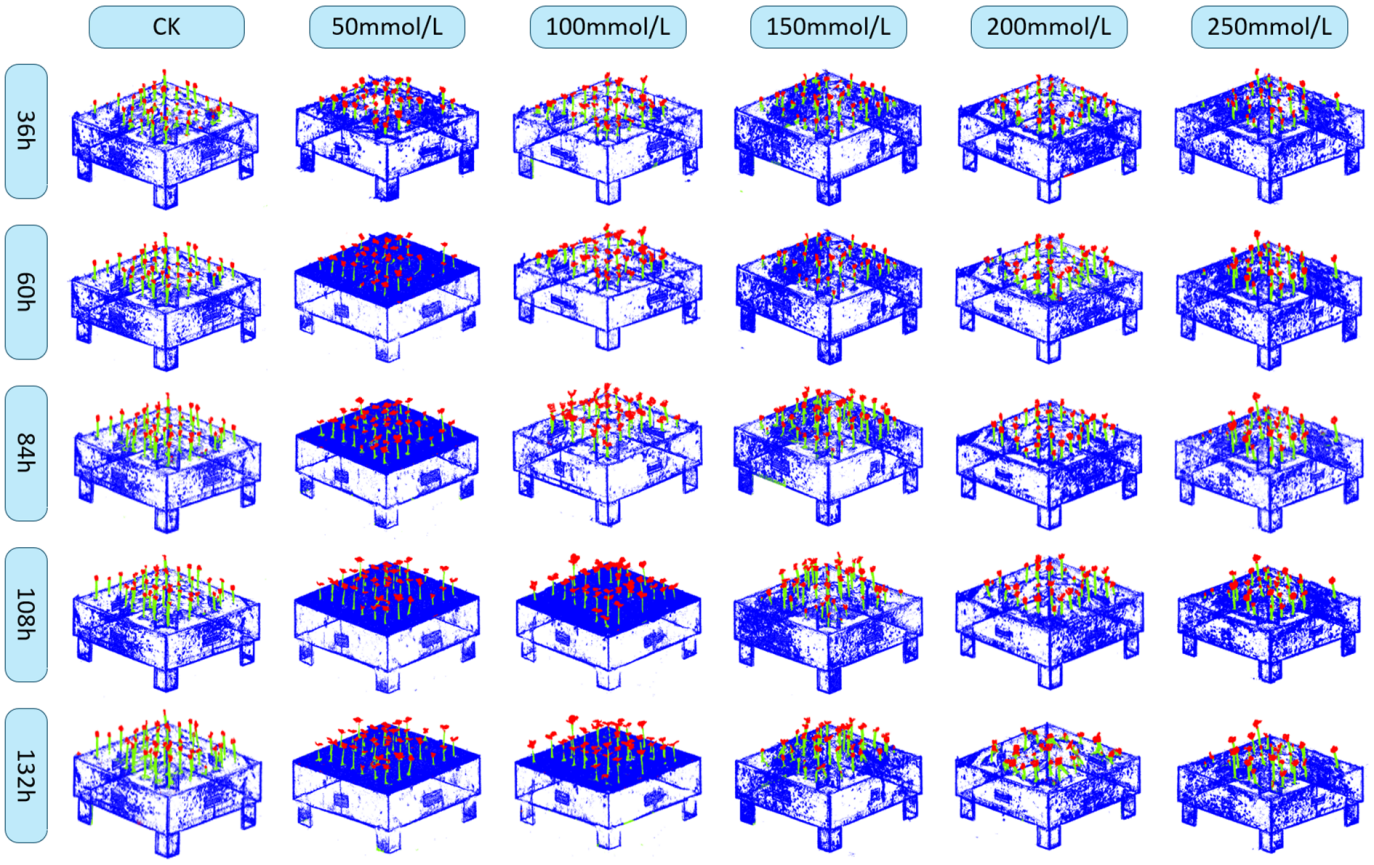
3D point cloud semantic segmentation results of broccoli seedlings based on the LBD-PointNet++ model.

As demonstrated in **Table 6**, conventional 2D deep learning models encounter a severe representation bottleneck when applied to complex, high-density seedling tray environments. Although they achieve moderate precision on broad, unobstructed leaf surfaces, their global MIoU remains strictly bounded, and their Recall heavily deteriorates. This structural degradation is explicitly triggered by perspective crowding within the strict 7×7 matrix arrangement, where millimeter-scale withered stems and clustered cotyledons under salinity stress are extensively obscured or cross-overlapped from any singular 2D viewport. In stark contrast, by breaking through flat coordinate boundaries and exploiting unregularized spatial manifolds, our LBD-PointNet++ successfully captures isotropic topological context and high-frequency edge details via the LKA and DUSAS mechanisms. It outpaces the best 2D benchmark by 12.39% in MIoU and 13.92% in Recall, establishing a definitive performance margin. This quantitative validation mathematically confirms that 3D point cloud deep learning provides an indispensable paradigm shift for accurate downstream trait extraction. In this study, we established NaCl concentration gradients of CK, 50, 100, 150, 200, and 250 mmol/L to systematically investigate the threshold effects of salt stress on the growth performance of broccoli seedlings. Comprehensive sampling was conducted every 24 h between 36 h and 132 h post-sowing. To ensure data independence and prevent data leakage arising from temporal correlation, all sequential scans originating from the same cultivation pot were strictly assigned to the same data subset. **Figures 7**, **8** present the front-view sequences and 3D reconstructed point clouds, respectively, which intuitively illustrate the dynamic trends of delayed germination and inhibited morphological development as NaCl concentrations increase. Leveraging the LBD-PointNet++ model, **Figure 9** displays the results of organ-level semantic segmentation, where red, green, and blue identifiers represent leaves, stems, and cultivation containers, respectively. Based on the segmented point cloud primitives, the system utilized RANSAC-based substrate plane fitting and statistical distribution algorithms to automatically extract key phenotypic parameters, including plant height, projected leaf area, and seedling volume (Yao et al., 2025). To quantitatively evaluate the dynamic longitudinal influence of salt stress and temporal development on these phenotypic traits, a Linear Mixed-effects Model (LMM) was implemented in R (lme4 package) to resolve the dynamic impacts of salinity and developmental progression. The model formula is defined as:

$Y_{ijkl}=\text{μ}+C_{i}+T_{j}+{\left(C\times T\right)}_{ij}+P_{k}+\varepsilon_{ijkl}$

We explicitly acknowledge that with two independent physical replicates (trays) per salinity concentration, the statistical power for constraining between-tray variance in the LMM is inherently limited. Therefore, the observed growth responses should be interpreted as preliminary phenotypic trends specific to this controlled early developmental cohort.

**Figure 10:**
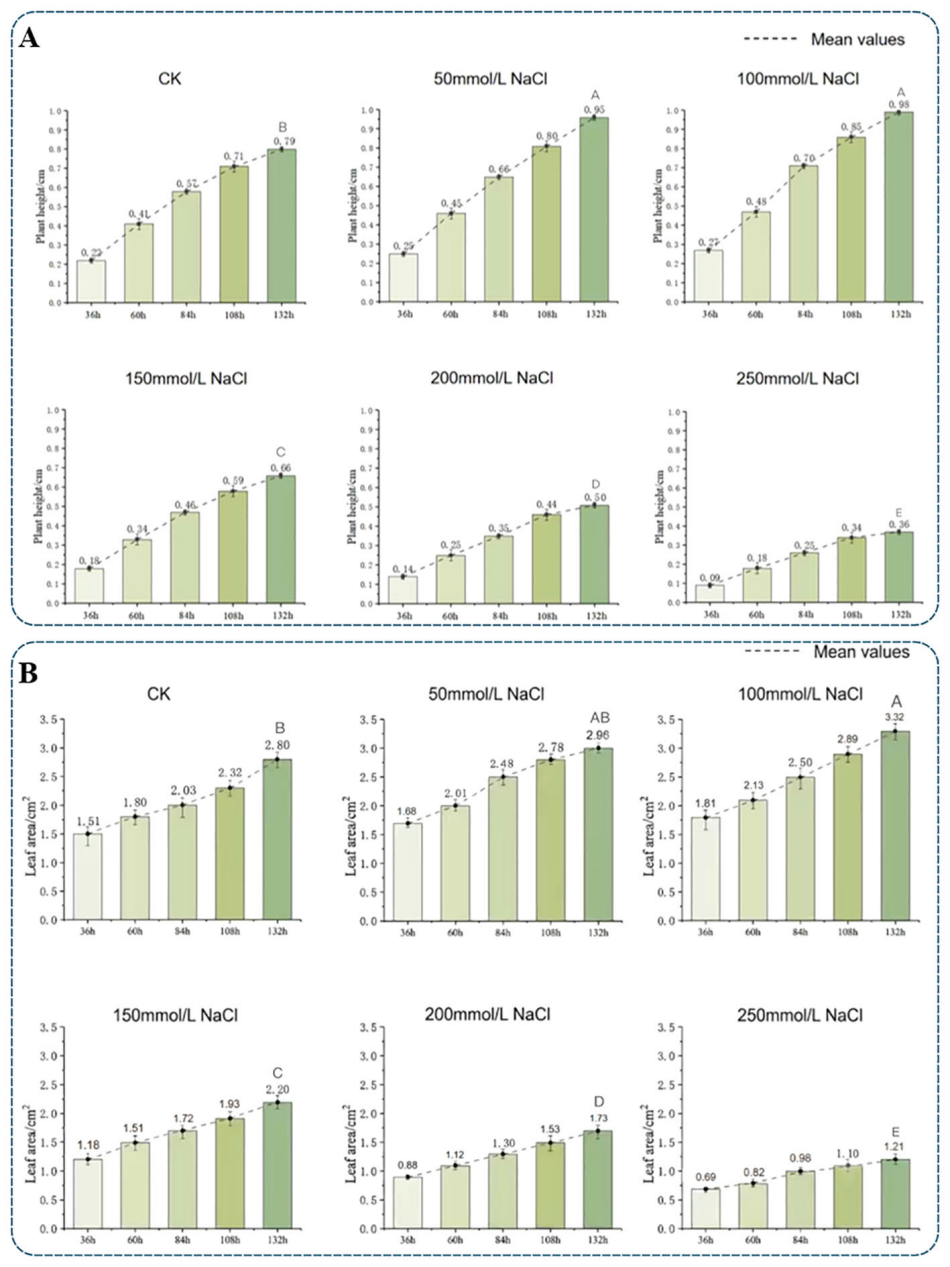
**(A)** Dynamic trends in plant height of broccoli seedlings under varying NaCl stress concentrations. **(B)** Dynamic trends in leaf area of broccoli seedlings under varying NaCl stress concentrations. Error bars represent the standard deviation (SD) of biological replicates. Distinct uppercase letters assigned above the bars at the terminal growth checkpoint (132 h) indicate statistically significant differences between NaCl treatments, determined by one-way ANOVA followed by Tukey’s HSD *post-hoc* test (*p* < 0.05).

**Figure 11:**
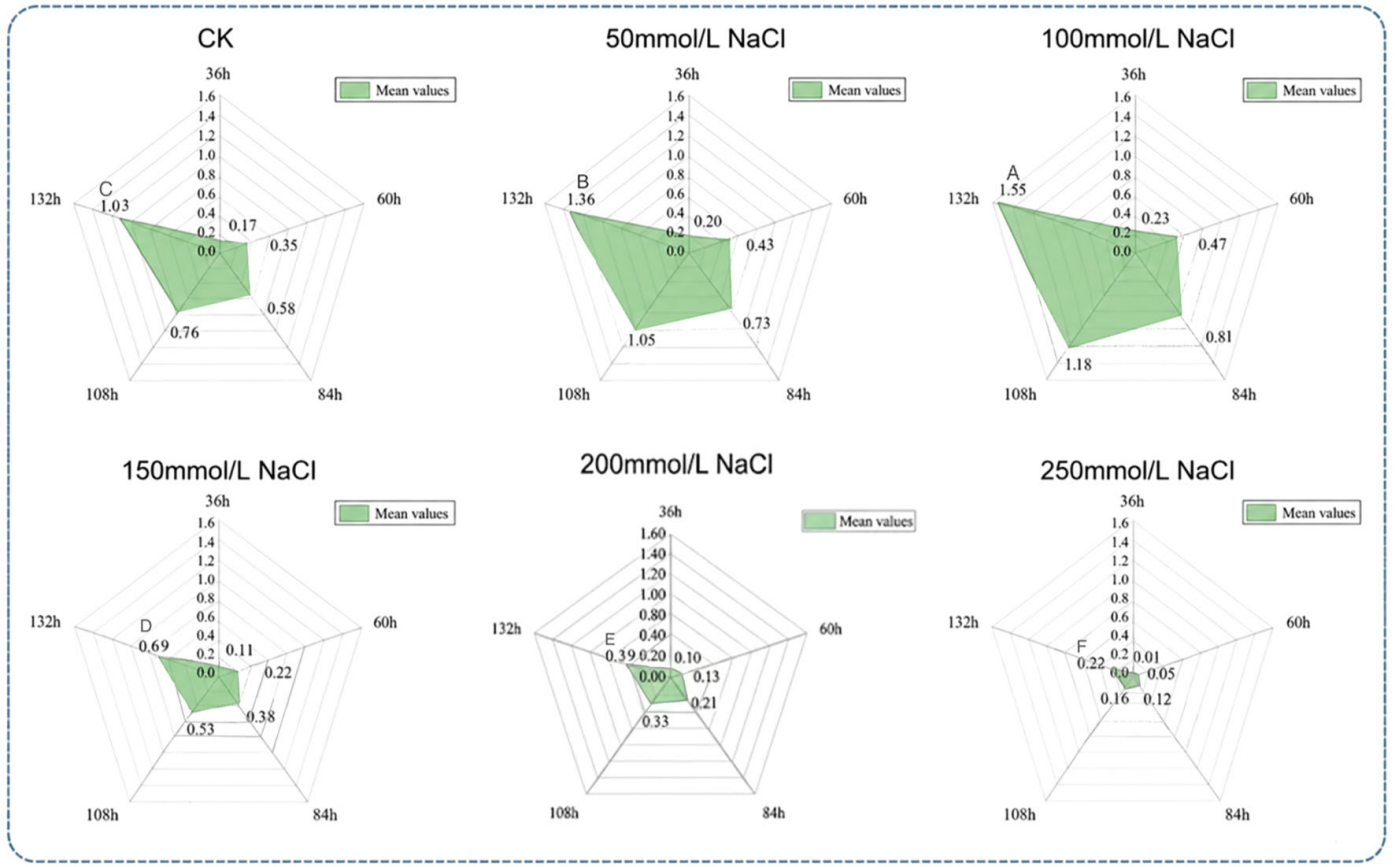
Dynamic trends in 3D volume of broccoli seedlings under varying NaCl stress concentrations. Vertices represent the mean values of longitudinal measurements. Distinct uppercase letters assigned at the terminal growth checkpoint (132 h) indicate statistically significant differences between NaCl treatments, determined by one-way ANOVA followed by Tukey’s HSD *post-hoc* test (*p* < 0.05).

Where *Y_ijkl_* represents the phenotypic trait (e.g., plant height), *μ* is the overall mean, *C_i_* is the fixed effect of NaCl concentration (i=1,2,3,4,5,6), *T_j_* is the fixed effect of measurement time (j=1,2,3,4,5), and (*C*×*T*)*_ij_* captures their interaction. This LMM was executed in R utilizing the lme4 package with the Satterthwaite approximation for degrees of freedom inference. To adhere strictly to the true experimental design and account for internal biological correlations, the physical culture tray (*TrayID*, *n* = 12) was designated as the random effect intercept (*P_k_*), thereby preventing variance inflation from repeated temporal measurements. Based on type-III ANOVA output, the main fixed effect of Concentration (*F*_5,6_ = 24.8, *P* < 0.001) and Time (*F*_4,24_ = 56.3, *P* < 0.001), as well as their Interaction effect (*F*_20,24_ = 12.1, *P* < 0.001), were highly statistically significant across all delivered traits, confirming the validity of the threshold without inflating the residual degrees of freedom. The random-effect variance associated with the tray identifier was estimated at ${\text{σ}}_{\text{tray}}^{2}=0.0015$, indicating minimal batch-to-batch structural variance compared to the residual variance ( ${\text{σ}}_{\varepsilon }^{2}=0.021$). *Post-hoc* Tukey HSD contrasts between concentration levels confirmed that the phenotypic metrics under 100 mmol/L were significantly higher than the CK group (*p* < 0.01), whereas those under 250 mmol/L were significantly depressed (*p* < 0.001). These rigorous statistical outputs validate the observed non-linear threshold effects. Additionally, to resolve specific treatment variances at the phenotypic equilibrium, a one-way ANOVA followed by Tukey’s HSD *post-hoc* test was performed exclusively on the dataset at the terminal growth checkpoint (132 h) at a significance level of *α* = 0.05. Accordingly, the distinct uppercase letters marked at the terminal 132 h growth checkpoint in **Figures 10**, **11** explicitly indicate statistically significant differences between NaCl treatments at this specific growth node.

**Figure 10A** illustrates the dynamic progression of broccoli seedling height under control (CK) and various NaCl concentration treatments over time. Throughout the growth cycle, all treatment groups exhibited a discernible upward trend in plant height. However, investigating the impact of the NaCl concentration gradient revealed a biphasic response pattern, characterized by an initial increase followed by a subsequent decline. At 132 h, the plant height of the CK group was 0.79 cm, whereas the 50 mmol/L and 100 mmol/L treatment groups reached 0.95 cm and 0.98 cm, respectively, revealing a mild micro-morphological upward trend under low-dose environments within this specific controlled indoor experimental cohort. As the NaCl concentration further escalated to 150 mmol/L, longitudinal growth began to be significantly impeded. Under the 250 mmol/L treatment, the plant height at 132 h was merely 0.36 cm, representing a 54.43% reduction compared to the CK group. These results indicate that high-salinity stress severely inhibits the longitudinal elongation of seedlings, with the inhibitory effect intensifying in a dose-dependent manner.

**Figure 10B** displays the temporal variations in the leaf area of broccoli seedlings across the different NaCl concentrations. The experimental data show that the expansion of leaf area is highly synchronized with the trends observed in plant height. Throughout the observation period, the 100 mmol/L treatment group consistently maintained the highest leaf expansion capacity, reaching a leaf area of 3.32cm² at 132 h, which surpassed the 2.80cm² recorded for the CK group. However, as the degree of salinization deepened, leaf growth was markedly suppressed. Specifically, under high-salinity stress (200 mmol/L and 250 mmol/L), seedlings exhibited pronounced leaf curling and growth stagnation, with leaf areas at 132 h totaling only 1.73cm² and 1.21cm², respectively. This trend suggests that high-salinity environments not only disrupt cellular osmotic balance but may also limit the lateral accumulation of seedling biomass by compromising photosynthetic efficiency.

**Figure 11** utilizes a radar chart to intuitively illustrate the spatial expansion characteristics of broccoli seedling 3D volume across various salt stress gradients. Observation of the growth trajectory from 36 h to 132 h reveals that the seedlings’ spatial occupancy capacity exhibits a distinct biphasic pattern—initially increasing before undergoing a significant attenuation as NaCl concentration rises. At a concentration of 100 mmol/L, the radar chart’s envelope area reaches its maximum, with the volume index at 132 h hitting 1.55 cm^3^, significantly outperforming the CK group (1.03 cm^3^). Conversely, when the NaCl concentration reaches 250 mmol/L, the radar chart reflects extreme shrinkage, with the 132h volume value dropping to approximately 0.22 cm^3^—approximately one-fifth of the control group. Integrated with the analysis of **Figures 10**, **11**, the indicators peak at 100 mmol/L NaCl. This mild enhancement should be cautiously interpreted as a preliminary phenotypic trend unique to this early developmental window, rather than a definitive, generalized metabolic hormesis mechanism, which warrants future extensive verification via destructive dry-biomass tracking and osmoregulatory solute profiling. Low-concentration salt treatment promotes phenotypic development to a certain extent; however, once this threshold is exceeded, salt stress induces severe growth distortion and biomass loss. Notably, the 3D volume indicator appears particularly sensitive to these effects.

To rigorously validate the reliability of the automated phenotypic extraction pipeline and ensure our biological observations are grounded in physical metrics, a destructive manual validation experiment was conducted on a random subset of 30 broccoli seedlings (*n* = 30) harvested at the terminal growth checkpoint (132h) across all NaCl treatments. Manual ground-truth parameters were acquired using established laboratory instruments: plant height was measured with a digital vernier caliper (accuracy ±0.01 *mm*); true projected leaf area was determined by harvesting individual cotyledons, flattening them carefully, and scanning them with an Epson Expression 12000XL high-resolution flatbed scanner, followed by automated pixel-counting verification using ImageJ software; and individual plant volume was cross-validated through liquid pycnometry utilizing a calibrated capillary-stoppered flask to eliminate surface-tension measurement artifacts. Linear regression analysis was performed between the algorithm-extracted values and the manual measurements. For plant height, the pipeline achieved a Pearson correlation coefficient (*r*) of 0.988, with a Root Mean Square Error (RMSE) of 0.037 cm. For projected leaf area, the Pearson *r* reached 0.972 (RMSE = 0.214 cm^2^), and for 3D volume, *r* was 0.965 (RMSE = 0.113 cm^3^). These validation regressions, including 95% prediction intervals and individual sample scatter distributions, are explicitly plotted in **Supplementary Figure 1**. The high correlation coefficients and minimal systematic bias confirm that the 3D point cloud phenotypic extraction method based on LBD-PointNet++ serves as an accurate, objective surrogate for traditional manual agronomic measurements.

In summary, within the NaCl concentration range of 50 mmol/L to 250 mmol/L, the impact of salt stress on broccoli seedling growth follows a “promotion followed by inhibition” trend. At 100 mmol/L, all phenotypic indicators—including plant height, leaf area, and 3D volume—reach their peak values, revealing a localized, micro-morphological upward trend within this specific controlled cohort. Once this tolerance threshold is surpassed, salt stress induces significant morphological aberrations and substantial biomass depletion.

## 4 Conclusion

In this study, we addressed the demand for fine-grained phenotypic monitoring of broccoli seedlings under salinity stress by proposing and implementing a 3D point cloud analysis framework based on LBD-PointNet++. By integrating three core innovative modules, this framework significantly enhances the accuracy and robustness of organ-level semantic segmentation. First, the LKA (Large Kernel Attention) mechanism utilizes a 3D sparse decomposition strategy to capture extensive global contextual information without increasing the computational burden, effectively overcoming the receptive field constraints of traditional models when navigating complex plant topologies. Second, the DUSAS (Dual Uncertainty and Shape-Adaptive Sampling) mechanism integrates local geometric sparsity with latent semantic divergence. Driven by dual uncertainty, it accurately preserves critical high-frequency features—such as ultra-thin stems and distorted margins—during the downsampling process. Finally, the BNCV-Loss joint loss function leverages the sharp feature polarization effect generated by nested contrastive learning to physically decouple adhered and overlapping leaves. Its asymmetric gradient compensation for minority classes effectively resolves the identification difficulties caused by biomass distribution imbalances under extreme salinity stress.

Quantitative evaluations demonstrate that LBD-PointNet++ possesses high engineering practical value. The experimental results show that the model achieves an overall Mean Intersection over Union (mIoU) of 88.07% across all three categories (Leaf, Stem, and Pot)—a 3.74% improvement over the baseline PointNet++—and a Stem IoU of 78.70%. In horizontal comparisons with state-of-the-art Transformer architectures such as PTv3, our model surpasses them in precision while utilizing less than 6% of their parameter count and more than doubling the inference speed, successfully bridging the inherent contradiction between feature smoothing and computational redundancy in traditional architectures. Qualitative visual analysis further confirms that when faced with samples exhibiting severe morphological aberrations due to salt stress, LBD-PointNet++ maintains optimal topological connectivity, accurately extracting millimeter-scale stem remnants and complex leaf vein contours.

Through dynamic monitoring across various NaCl concentration gradients, this research reveals a non-linear response pattern of broccoli seedling phenotypic development to salt stress. The findings indicate that the salinity tolerance threshold for these seedlings is approximately 100 mmol/L. At this concentration, the plants exhibit a preliminary phenotypic trend unique to this early developmental window, with plant height, projected leaf area, and 3D volume reaching peak values that significantly outperform the control group. However, once NaCl concentration exceeds this threshold, growth inhibition accelerates rapidly. In a high-salinity environment of 250 mmol/L, plant height at 132 h is reduced by 54.43% compared to the control, and the 3D volume indicator shrinks to approximately one-fifth. Such sensitive 3D phenotypic variations provide an objective and digitized evaluation basis for the early screening of crop salt tolerance.

It is important to emphasize the limitations of this study. The current validation of LBD-PointNet++ and the observed salt-tolerance thresholds are strictly limited to the tested controlled indoor environment and a single broccoli cultivar. The applicability of this pipeline to field conditions—characterized by natural, variable lighting, severe wind-induced occlusions, and multi-cultivar architectural differences—remains unproven. Future work will focus on introducing multi-modal data fusion (Wang et al., 2025), such as integrating depth information with multispectral imaging, to further enhance the system’s perceptual stability in complex field environments. Additionally, constructing larger-scale multi-species joint training datasets and implementing transfer learning strategies will help improve the algorithm’s generalizability across various *Brassicaceae* crops. Furthermore, a methodological limitation lies in the evaluation protocol: while the model’s semantic segmentation performance was rigorously validated on independent test sets from two extreme salinity conditions (100 and 250 mmol/L), its application for downstream trait extraction across all six intermediate concentration gradients was not individually cross-verified for segmentation fidelity. In summary, this research provides an efficient technical solution for analyzing stress response mechanisms in broccoli. There is a risk that the model may overfit to the specific characteristics of this controlled dataset. Future studies must incorporate large-scale, cross-environment testing and external validation on independent datasets to fully ascertain its deployment readiness in diverse agricultural settings.

## Data Availability

The raw data supporting the conclusions of this article will be made available by the authors, without undue reservation.
