# Supplementary material for: LBD-PointNet++: a point cloud segmentation network for phenotypic trait extraction of broccoli seedlings
Source: Front Plant Sci. 2026 Jul 31;17:1835285. doi: 10.3389/fpls.2026.1835285 (PMC13473401; doi:10.3389/fpls.2026.1835285)
Supplement: Supplementary file 1 [file DataSheet1.pdf]

The Table S1 provides the complete per-class metrics corresponding to the aggregated results visualized in Figure 5 of the main manuscript, allowing for a detailed comparison of performance across all classes and methods.

Table S1 Exhaustive breakdown of semantic segmentation performance across individual plant organs for mainstream 3D deep networks under strict 5-fold tray-disjoint cross-validation

Index	Part	IoU	Precision	Recall	F1-Score	OA
**PointNet++**	Pot	96.07	97.53	98.47	98.00	93.85
	Leaf	83.07	91.48	90.04	90.75	
	Stem	73.85	86.52	83.45	84.96	
	Mean	84.33±0.68	91.84±0.62	90.65±0.75	91.24±0.70	
**RandLA-Net**	Pot	95.81	97.48	98.24	97.86	92.20
	Leaf	82.38	90.72	89.96	90.34	
	Stem	70.43	85.34	80.12	82.65	
	Mean	82.87 ± 0.75	91.18 ± 0.69	89.44 ± 0.82	90.28 ± 0.76	
**PTv3**	Pot	96.99	98.11	98.84	98.47	96.33
	Leaf	87.17	94.27	92.05	93.15	
	Stem	76.27	91.04	82.46	86.54	
	Mean	86.81 ± 0.58	94.47 ± 0.52	91.12 ± 0.64	92.72 ± 0.55	
LBD-	Pot	97.52	98.22	99.27	98.74	

PointNet++	Leaf	88.00	94.46	92.79	93.62	97.35
	Stem	78.70	89.78	86.45	88.08	
	Mean	88.07±0.51	94.15±0.38	92.84±0.42	93.48±0.35	

The Figure S1 and Table S2 provide the comprehensive per-sample datasets and linear regression assessments for the validation seedling cohort ($n = 30$), corresponding to the metric accuracy validation described in the main manuscript to permit a fully transparent evaluation of absolute metric calibration and geometric precision across key phenotypic traits.

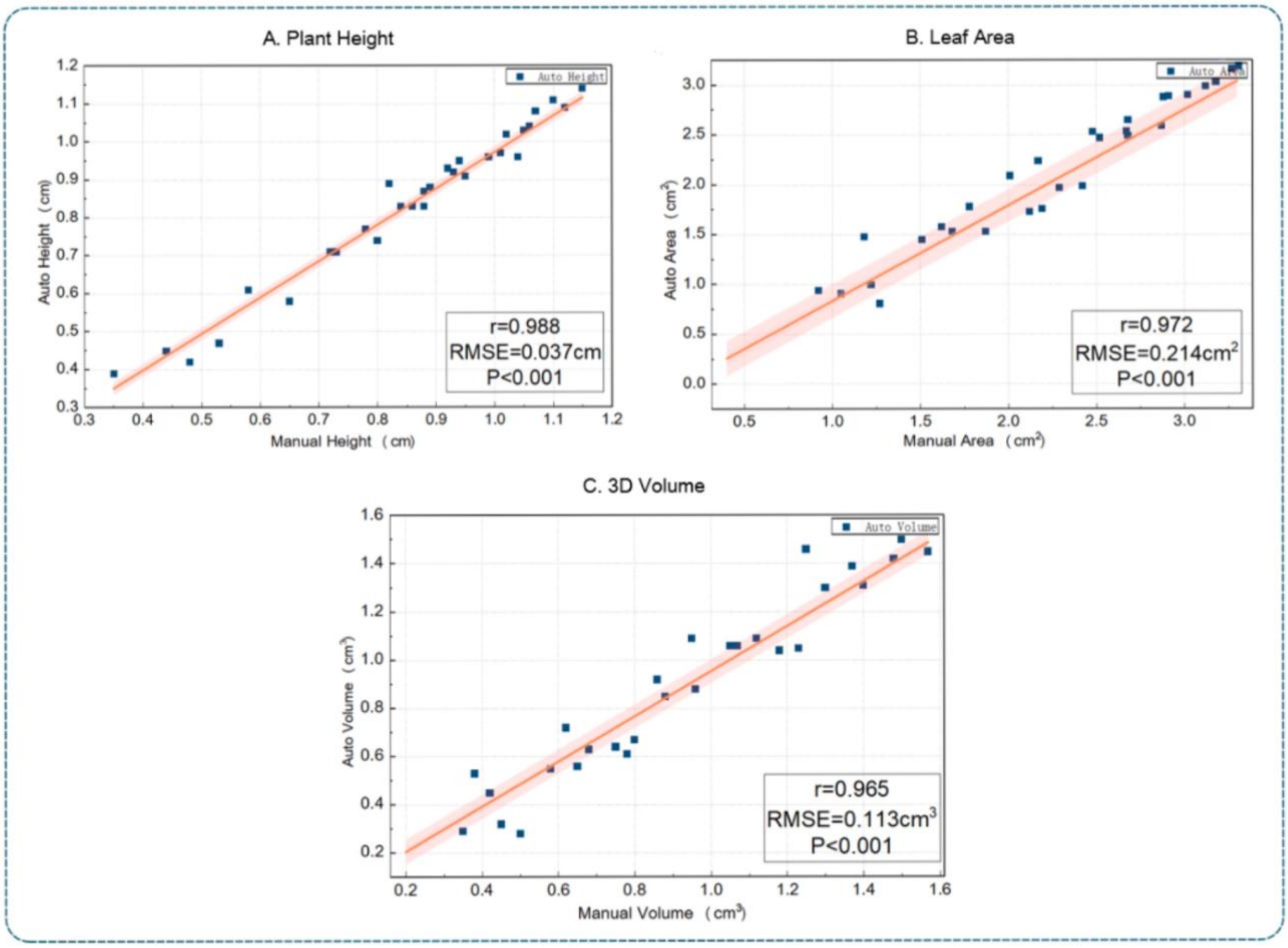

Figure S1 Correlation and linear regression analysis between manual ground-truth measurements and LBD-PointNet++ automated estimations for broccoli seedling phenotypic traits ($n = 30$)

Table S2. Exhaustive dataset of paired phenotypic metrics comprising manual ground-truth measurements and LBD-PointNet++ algorithmic predictions for the validation broccoli seedling cohort ($n = 30$)

Sample ID	Manual Height (cm)	Auto Height (cm)	Manual Area (cm2)	Auto Area (cm2)	Manual Volume (cm3)	Auto Volume (cm3)
S01	0.88	0.83	2.48	2.53	1.07	1.06
S02	0.92	0.93	2.67	2.54	1.23	1.05
S03	0.95	0.91	2.88	2.88	1.37	1.39
S04	1.02	1.02	3.02	2.90	1.50	1.50
S05	1.05	1.03	3.18	3.03	1.57	1.45
S06	1.10	1.11	3.27	3.16	1.63	1.41
S07	1.15	1.14	3.31	3.19	1.68	1.58
S08	0.86	0.83	2.52	2.47	0.86	0.92
S09	0.82	0.89	2.29	1.97	0.80	0.67
S10	0.78	0.77	2.12	1.73	0.75	0.64
S11	0.73	0.71	1.87	1.53	0.68	0.63
S12	0.65	0.58	1.68	1.53	0.58	0.55
S13	0.58	0.61	1.51	1.45	0.50	0.28

S14	0.44	0.45	1.18	1.48	0.42	0.45
S15	0.35	0.39	0.92	0.94	0.35	0.29
S16	0.48	0.42	1.05	0.91	0.38	0.53
S17	0.53	0.47	1.22	1.00	0.45	0.32
S18	0.72	0.71	1.27	0.81	0.62	0.72
S19	0.80	0.74	1.62	1.58	0.65	0.56
S20	0.88	0.87	1.78	1.78	0.78	0.61
S21	0.93	0.92	2.01	2.09	0.88	0.85
S22	0.99	0.96	2.19	1.76	0.95	1.09
S23	1.04	0.96	2.68	2.49	1.12	1.09
S24	1.07	1.08	2.87	2.59	1.25	1.46
S25	1.12	1.09	3.12	2.99	1.40	1.31
S26	1.01	0.97	2.91	2.89	1.30	1.30
S27	0.94	0.95	2.68	2.65	1.18	1.04
S28	0.89	0.88	2.42	1.99	1.05	1.06
S29	0.84	0.83	2.17	2.24	0.96	0.88
S30	1.06	1.04	3.22	3.28	1.48	1.42

Mean	0.85	0.84	2.27	2.15	0.98	0.94
St. dev	0.21	0.21	0.73	0.75	0.40	0.40
